# Recent advances and trends in magnetic nanoparticle-assisted aptasensors for foodborne bacteria monitoring: applications, challenges, and updates

**DOI:** 10.1016/j.fochx.2025.103156

**Published:** 2025-10-16

**Authors:** Narges Kiani-Salmi, Behnam Bahramian, Reza Abedi-Firoozjah, Alireza Ebrahimi, Arezou Khezerlou, Milad Tavassoli, Ali Ehsani

**Affiliations:** a Department of Food Science and Technology, Faculty of Nutrition and Food Sciences, Tabriz University of Medical Sciences, Tabriz, Iran; b Student Research Committee, Tabriz University of Medical Sciences, Tabriz, Iran; c Student Research Committee, Kermanshah University of Medical Sciences, Kermanshah, Iran; d Nutrition Research Center, Tabriz University of Medical Sciences, Tabriz, Iran; e Department of Nutrition, Faculty of Health and Nutrition Sciences, Yasuj University of Medical Science, Yasuj, Iran

**Keywords:** Food-borne bacteria, Sensor, Aptamer, Magnetic nanoparticles, Synthetic techniques

## Abstract

Foodborne illnesses are a serious threat to public health. This study reviews recent advances in magnetic nanoparticle-based aptasensors for monitoring foodborne bacteria, focusing on their applications, challenges, and future prospects. We analyze the latest developments in magnetic nanoparticle-based aptasensors in optical (colorimetric, fluorescence, surface-enhanced Raman scattering), electrochemical, and other sensors for the detection of foodborne bacteria. We review the application of magnetic nanoparticles (MNPs) in biosensor structures for various purposes, including signal generation and processing, purification, immobilization of target biomarkers, and bacterial isolation. Various challenges are discussed, such as increasing the affinity of aptamers, commercialization barriers, multiplex detection of bacteria, environmental concerns, and MNPs synthesis methods. To advance the field of magnetic nanoparticle-based aptasensors, future research should focus on optimizing different methods of magnetic nanoparticle synthesis, application of artificial intelligence and machine learning, integration of various diagnostic methods, interdisciplinary collaboration, development of a portable diagnostic device, and overcoming commercialization challenges.

## Highlights

• Recent advances in various magnetic nanoparticle aptasensors were highlighted.
• The advantages and disadvantages of different methods for the synthesis of magnetic nanoparticles were reviewed.
• Current challenges, technical barriers, and further development of these sensors were reviewed.
• Aptamers can bind to magnetic nanoparticles and shorten the detection time of foodborne pathogens.

## 1 Introduction

**Scheme 1:**
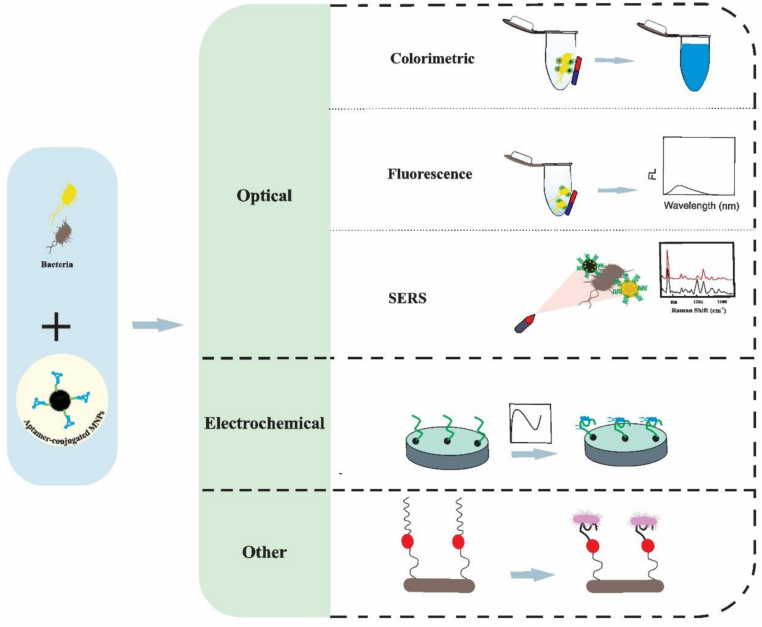
Overview of magnetic nanoparticle-assisted aptasensors for foodborne bacteria monitoring.

Contaminated food causes an estimated 600 million cases of foodborne disease and 420,000 deaths worldwide annually (Bhaskar, 2017). Although this problem is most often associated with low- and middle-income countries, high-income countries also face significant challenges. In the United States, foodborne illnesses caused by pathogenic bacteria account for more than 9.4 million cases annually (Riu & Giussani, 2020). One of the main responsibilities of the food industry is to deal with the challenges of pathogenic microorganisms. *Escherichia coli* (*E. coli*(, *Salmonella*, *Campylobacter*, and *Listeria* are among the most common causes of foodborne disease in humans. Their presence in various environments and their ability to adapt to diverse conditions make early and rapid detection essential to ensure food safety and quality (Cossettini et al., 2022, Rahimizadeh et al., 2023). Culture-based methods are sensitive and reliable, but they require significant expertise because of their multiple steps and time-consuming procedures. Enzyme-linked immunosorbent assays (ELISA) and advanced molecular techniques, such as polymerase chain reaction (PCR), have accelerated the detection process. However, they require sophisticated, precise equipment and skilled operators (Hao et al., 2019). Hence, there is an urgent need for more efficient, cost-effective, and reliable techniques for monitoring contamination in food and water. Today, a wide range of novel techniques has been developed for detecting pathogens. One of the new diagnostic technologies is the DNAFoil method. The advantages of this method include a) performing sample preparation steps without the need for centrifuges and spin columns, b) DNA amplification without the need for trained personnel with the help of thermal cycling and cold chain, and c) the possibility of immediate, on-site, and cost-effective diagnosis without the need for expensive and advanced laboratory equipment (El Sheikha, 2021). HRM is a novel molecular technique. This method is based on the principle that different double-stranded DNA molecules have different melting points. HRM uses fluorescent probes or dyes to examine changes in the shape of the curve, ultimately leading to the identification and detection of various pathogens with high accuracy and sensitivity. The advantages of this method include high sensitivity, speed, and specificity. Poor primer design can lead to false results. Also, the uniformity of the temperature of the device is of great importance (Liu et al., 2023). Biosensors have emerged as a powerful tool for the rapid and accurate detection of foodborne pathogens, addressing the growing concern over food safety. These devices utilize biological recognition elements, such as aptamers (Hu et al., 2023), enzymes (Kurbanoglu et al., 2020), antibodies (Chen et al., 2024), or DNA probes (Barrias et al., 2024), to specifically identify foodborne pathogens like *Salmonella*, *E. coli*, and *Listeria*. Once the pathogen is detected, the biosensor's transducer converts the biological interaction into a measurable signal, such as an electrical current or optical change. The advantages of biosensors in food safety applications include their high sensitivity, portability, and the ability to provide real-time results, making them invaluable for both routine monitoring and emergency response in food processing and distribution (Bhatia et al., 2024). With further advancements in biosensor technology, these tools are expected to enhance food safety protocols, reduce the risk of contamination, and ensure public health protection. Therefore, the need to develop more sensitive, faster, cost-effective, and portable aptasensors capable of detecting bacteria in situ is felt more than ever today. Aptasensors, which utilize aptamers (single-stranded RNA or DNA oligonucleotides) to detect specific targets, face several limitations. These include lower sensitivity for detecting very low concentrations of analytes, susceptibility to degradation over time, particularly in harsh environments such as extreme pH, temperature, or ionic strength, and potential non-specific binding to other substances, which can lead to false positives. Additionally, aptamers may lose their functionality after repeated use, requiring complex regeneration processes (Geleta, 2022). One of the most widely used biosensing platforms is the lateral flow assay (LFA). LFAs have attracted much attention due to their low cost, simple and visual results, in situ response, and simple process. The overall process of this method is based on the interaction between the antibody and antigen or the hybridization between the DNA probe and the target. The main limitation of LFAs is related to their low sensitivity when the test is recorded by the naked eye (Sohrabi et al., 2022). Magnetic nanoparticles (MNPs) are a group of nanoparticles that uniquely enhance the selectivity and efficiency of biosensors. MNPs possess properties such as large specific surface area, magnetic enrichment capabilities, low toxicity, and high biomolecule binding rate, which have led to their selection for various biological investigations (Wang et al., 2024). In recent years, aptamer-modified magnetic nanomaterials have gained widespread use in food safety applications. This novel technology has played a crucial role in the detection of pathogenic bacteria (Kang et al., 2023), the identification of antibiotics (Guo et al., 2024), the detection of pesticides, the identification of biotoxins, and the detection of adulteration (Razzaghi et al., 2025). MNPs enhance aptasensor performance in several key ways. They enhance sensitivity by amplifying the signal, enabling more efficient target capture and stronger detection responses. The magnetic properties of MNPs enhance the signal by facilitating the collection of aptamer-target complexes, thereby reducing background noise and improving the signal-to-noise ratio (Herr et al., 2006). MNPs also concentrate on low-abundance targets, making it easier to detect trace amounts (Khodadadi et al., 2018). In addition, they help to improve aptamer stabilization, increase their shelf life, and improve resistance to environmental factors (Huang et al., 2023). Magnetic separation leads to the separation of complexes, reuse, and increased regeneration efficiency. These advantages make aptasensors more reliable and sensitive for wide applications. In the present study, we aim to review the progress and application of magnetic nanoparticle-based aptasensors for monitoring foodborne bacteria. For this purpose, we first review the different methods of synthesis of MNPs and provide a brief overview of aptamers and their properties. We also show how combining aptamers with MNPs enables the fabrication of diagnostic aptasensors. We review the types of magnetic nanoparticle-based aptasensors, including optical types, such as colorimetric, fluorescence, and surface-enhanced Raman scattering (SERS), and electrochemical types, along with other aptamer-based methods, with an emphasis on studies in the past few years (2020–2025) (Scheme 1). In the final section, current challenges and future directions in the application of these aptasensors in monitoring foodborne pathogens have been discussed to provide researchers in this field with innovative and practical insights for the design and development of magnetic nanoparticle-based aptasensors for the detection of foodborne bacteria.

## 2 Methods

Text search was performed using various databases such as ScienceDirect, PubMed, Google Scholar, and Web of Science. The keywords (“magnetic nanoparticles” OR “MNPs” OR “Fe₃O₄”), AND (“aptasensor” OR “aptamer-based biosensor” OR “aptamer”) AND (“bacteria” OR “foodborne bacteria” OR “foodborne pathogen” OR “*E. coli*” OR “*Salmonella*” OR “*Listeria*” OR “*Campylobacter*”) AND (“detection” OR “monitoring” OR “biosensing”) were used for searching.

For the literature search, the inclusion criteria were: 1) foodborne bacteria, 2) MNPs, 3) aptasensors used for detection, and 4) articles published in the last five years (2020–2025). The exclusion criteria were as follows: 1) microorganisms other than bacteria, 2) magnetic beads and metal nanoparticles, 3) sensors that do not use aptamers in their structure, and 4) articles published before 2020. Articles that met the criteria for inclusion in the study were thoroughly reviewed, and their data were extracted.

## 3 Fabrication and properties of magnetic nanoparticles

Various methods have been developed for the synthesis of MNPs. These methods are divided into three general categories: chemical, physical, and biological methods. Details of these methods are provided in the Supplementary Section. Additionally, the advantages and disadvantages of these synthesis approaches are summarized in Table S1.

## 4 Aptamers

Synthesis of monoclonal antibodies is expensive, laborious, and time-consuming. Other disadvantages include temperature sensitivity and imprecision (in the presence of small amounts of microorganisms in the environment). Aptamers can be suitable alternatives to antibodies (22). (Córdova-Espinoza et al., 2024). Aptamers are oligonucleotide sequences (RNA or DNA) that bind with high affinity and specificity to their targets. Aptamers have a variety of structures due to intramolecular interactions and complementary pairing of nucleic acids. These oligonucleotide sequences bind to target molecules using electrostatic interactions, van der Waals forces, π-π stacking interactions, and hydrogen bonding (Yifei et al., 2023). Aptamers are used in the construction of biosensors due to their unique properties, such as high affinity and sensitivity, high stability, bioavailability, and compact size (Rajkumari et al., 2024). However, the non-portable design and low sensitivity have led to the limited application and use of aptamers in real samples. Several methods have been reported to improve the performance of aptamers, such as end fixation, post-SELEX, chemical modification, etc. (Mingwei et al., 2024). Food pathogens can be detected directly or indirectly using aptamer-based sensors, based on aptamer binding to specific targets and converting the output into a detectable signal (by the *transducer*). When food pathogens bind to their specific aptamers, the spatial composition and structure of the aptamer are changed, which helps separate the pathogen from the interfering substances in the tested sample. This process leads to the elimination of complex matrix interference and improved detection sensitivity (Yang et al., 2023b). In recent years, extensive studies have been conducted on the application of nanoparticles-assisted aptasensors, which can be referred to as metal nanoparticles, quantum dots, metal oxides, and MNPs integrated with aptamers (Qin et al., 2024).

## 5 Properties and integration of aptamer and magnetic nanomaterials

**Fig. 1:**
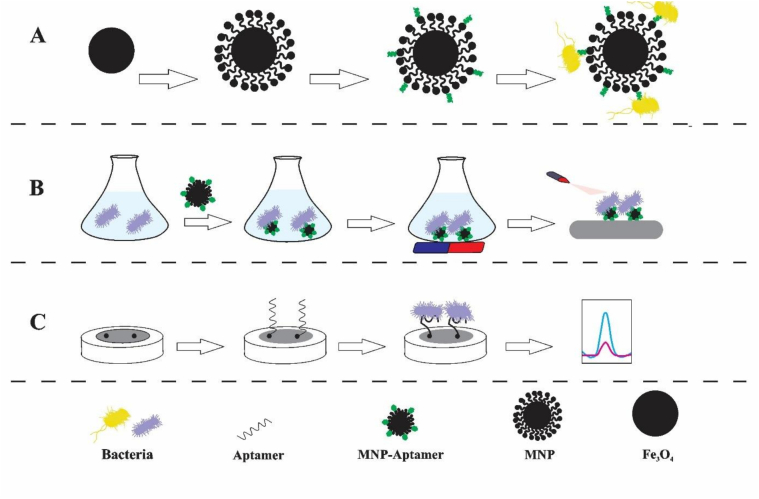
Applications of nanomaterials in biosensor structures (a) immobilization of target biomarkers, (b) separation, and (c) generation, conversion, and amplification of signals.

**Fig. 2:**
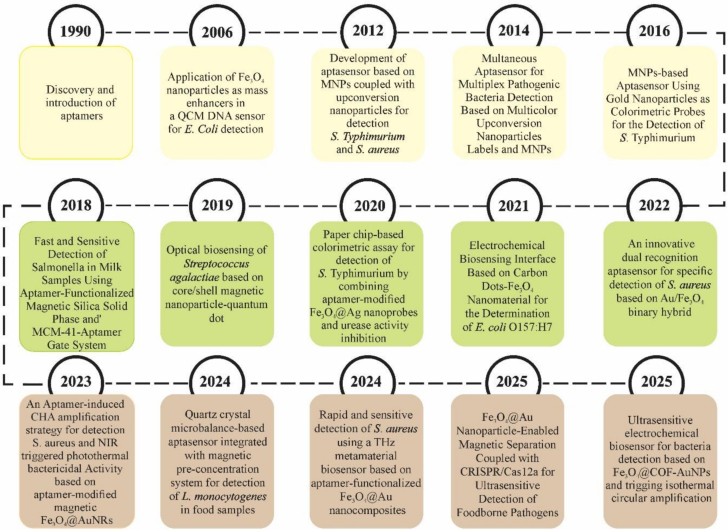
Innovation timeline of magnetic nanoparticle-assisted aptasensors for foodborne bacteria monitoring.

The application of nanomaterials in the structure of biosensors is carried out with several goals, such as production, transformation, and amplification of signals, purification, immobilization of target biomarkers, and isolation. It leads to fast response time, improved performance, a smaller amount of analytes, and good reproducibility and stability of nanosensors (Zi-yue et al., 2020). MNPs can enhance and amplify signals by directly contacting the electrode surface, coating the electrode surface with a thin layer, and moving and translocating active redox species towards the electrode, leading to improved selectivity and sensitivity of sensors (Hasanzadeh et al., 2015). MNPs are suitable for the purification and separation of compounds that are of great importance due to their environmental stability, cost-effectiveness, and selectivity. In addition to being separated by an external magnetic field, MNPs also affect liquid structures, which is why they are used in aqueous extraction and purification techniques. They can provide an active surface for the adsorption of biological macromolecules such as proteins, bacteria, and nucleic acids. One of the important advantages of nanomaterials is their high specific surface area, which allows the immobilization of large amounts of bioreceptor units (Holzinger et al., 2014). Torres-Cartas et al. synthesized a selective and efficient adsorbent by immobilizing aptamers on the surface of MNPs. In this study, SH-aptamer was covalently attached to the surface of vinylized MNPs, forming Apt-MNPs. This adsorbent was used for the removal of atrazine and showed satisfactory recovery and reusability (Torres-Cartas et al., 2023). One of the essential issues related to biosensors is the possibility of proper immobilization of biological markers on the transducer surface. The lack of directional immobilization of biological molecules can lead to the loss of biological reactions and catalytic activities. This process can, in turn, hinder electron transfer and reduce the electrochemical signal, resulting in reduced reproducibility, selectivity, and sensitivity of the system. The use of MNPs in biosensors increases the efficiency of analyte concentration and separation, and increases the kinetics. It improves the sensitivity and detection limit, and reduces the analysis time, resulting in improved selectivity and reproducibility (Fig. 1) (Carinelli et al., 2023). In terms of size, nanomaterials are similar to many biological macromolecules, such as proteins and nucleic acids. On the other hand, MNPs have unique magnetic properties that can be controlled by applying a magnetic field. MNPs are widely used in various fields. In the use of MNPs in the laboratory field, the surface of nanoparticles can be modified with various receptors or ligands and used to detect and isolate specific nucleic acids, proteins, viruses, etc. (Natalia et al., 2023). The application of MNPs in aptasensors is mostly introduced as a separator. MNPs are functionalized by various functional groups such as NH2, COOH, and OH. As a result, they find more modifications with conjugated aptamers (Kurup et al., 2021). MNPs are attached to aptamers to increase selectivity for MNP-based samples. This process combines Apt-MNPs with the sample and uses a magnetic field to separate the adsorbed analyte. This method has been widely used due to its many features, such as environmental compatibility, easy application, and high extraction efficiency (Özge et al., 2024). The large surface area, easy separation using magnets, and surface functionalization have led to the wide application of MNPs in the structure of biosensors. Also, the combination of MNPs with aptamers leads to the facilitation of electron transfer in electrochemical biosensors and the emergence of highly selective and sensitive biosensors (Milad Baghal et al., 2024). Apt-MNPs are used to identify proteins and small molecules. Biological samples do not have magnetic properties, so using MNPs is very suitable for sensitive detection without creating background signals (Farag et al., 2024; Urmann et al., 2017). As explained in Section 4, aptamers bind to their targets through specific molecular interactions. The use of nanomaterials and their combination with aptamers has improved the detection process in various fields (Li et al., 2021; Mahmoudpour et al., 2021; Rajkumari et al., 2024). Compared to traditional adsorbents, MNPs offer advantages, such as short diffusion routes, superparamagnetic properties, surface active areas, high surface-to-volume ratio, reusability of the sorbents, and low consumption of organic solvents. The advantages mentioned, along with the synthesis of functionalized MNPs with aptamers and the good selectivity of aptamers, are very suitable for extraction. Among different MNPs, iron oxides such as maghemite (γ-Fe_2_O_3_) and magnetite (Fe_3_O_4_) nanoparticles are the most used for extraction purposes (Natalia et al., 2023). For this purpose, they are modified at the 3′ and/or 5′ ends with enzymatic or electroactive labels and functionalized with appropriate moieties to stabilize them on magnetic substrates (Mattarozzi et al., 2022). A timeline summarizing 35 years of innovation in the field of magnetic nanoparticle-based aptasensors is also provided in Fig. 2. This table shows the progression from the introduction of aptamers and the initial application of magnetic nanoparticles to the fabrication of advanced nanocomposites. These nanocomposites enable magnetic separation through various optical and electrochemical processes, while advanced materials (COFs, metamaterials) and gene editing (CRISPR) allow for improved bacterial detection, ultimately leading to improvements in food safety. In the next section, we review recent advances in this field.

## 6 Magnetic nanoparticle-assisted aptasensors for foodborne bacteria monitoring

**Fig. 3:**
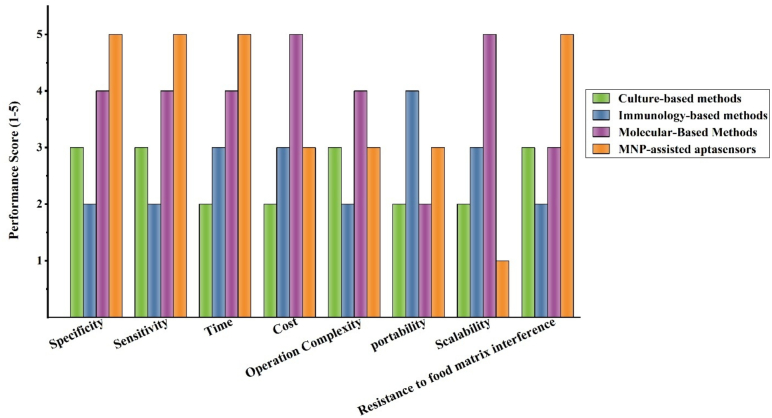
Comparison of the performance of different methods for detecting pathogenic bacteria. Scores are presented on a scale of 1 to 5, with 1 representing the lowest performance.

Food poisoning and infection caused by pathogenic bacteria are still one of the key causes of human mortality. Accurate and rapid monitoring of pathogenic bacteria is of great importance in reducing the risks of food contamination. As mentioned, methods such as culture-based methods, immunological-based methods, and molecular-based methods are the most widely used methods for bacterial detection. However, these methods are time-consuming, require trained personnel, and bulky equipment. The emergence of biosensors has overcome many of these problems. Table 1 provides a comparison between different methods, culture-based methods, immunological-based methods, molecular-based methods, and magnetic nanoparticle-assisted aptasensors for the detection of foodborne bacteria. The bar chart in Fig. 3 also examines the performance of four diagnostic methods in eight key parameters: Specificity, Sensitivity, Time, Cost, Resistance to food matrix interference, operation complexity, portability, and scalability. Scores are presented on a scale of 1 to 5, with 1 representing the lowest performance. In recent years, various types of magnetic nanoparticle-assisted aptasensors have been developed to detect foodborne pathogens, including optical, electrochemical, and other kinds of aptasensors.

**Table 1:** Comparison of different detection methods for foodborne bacteria**.**

Parameter	Culture-based methods	Immunology-based methods	Molecular-Based Methods	magnetic nanoparticle-assisted aptasensors
Specificity	Moderate Isolation of the target microorganism by selective media and biochemical tests	Low Probability of false positive results	High Using dedicated primers	High Use of specific aptamers on MNPs
Sensitivity	Moderate (not suitable for very high sensitivity) Limited sensitivity for low-abundance microbes	10^3^–10^4^ CFU/mL Less sensitive than PCR and biosensors	High 1 CFU/mL	Very high LOD <1 CFU/mL Due to the use of MNP and combined methods
Time	18–72 h Requires pretreatment and cultivation Long time	Relatively fast Usually, a few hours (e.g., ELISA about 2 hours in enriched samples)	High Some methods, such as ultrafast PCR, can be performed in as little as 3 to 6 h or less than 30 min.	Short detection time due to the use of MNP for rapid separation
Cost	Low Inexpensive, requires cheap equipment and materials	Moderate Cost of kits and reagents	Relatively high Requires expensive specialized equipment, although lower-cost portable devices are also being developed	Variable cost Using gold nanoparticles leads to a high cost Most iron or iron oxide-based methods are inexpensive 3
Resistance to food matrix interference	Moderate Relatively resistant Requires pre-enrichment and enrichment for the detection of some bacteria	Low Food matrix sensitive Disruption caused by food components such as fat and protein	Moderate Possibility of PCR inhibition by some food matrix components (e.g., carbohydrates, fats, etc.)	Highly resistant to complex food matrices
Operation Complexity	Moderate Need for trained personnel and laboratories	Low Requires basic training	High Need for trained personnel	Moderate Some methods require special equipment, such as fluorescence devices. But colorimetric methods are very simple.
Portable	Moderate Requires large equipment and a laboratory	High Not all methods are portable	Low Although mini-PCR devices have been developed, they require large equipment and a controlled environment to perform the method.	Moderate Some methods require laboratory equipment, but some platforms can be used on-site.

**Parameter**	**Culture-based methods**	**Immunology-based methods**	**Molecular-Based Methods**	**magnetic nanoparticle-assisted aptasensors**
Scalability	Low suitable for small samples	Moderate Suitable for medium-sized samples, but some kits can be used for larger quantities.	High Ability to test multiple samples simultaneously	Low Limited commercialization
Reference	(Canciu et al., 2021, Hameed et al., 2018, Jangid et al., 2025, Lee et al., 2015)	(Saroj et al., 2008, Wang & Salazar, 2016, Notermans and Wernars, 1991)	(Kim et al., 2023, Lee et al., 2021, Liu et al., 2022)	(Sadsri et al., 2020, Yan et al., 2023, Zhou et al., 2025)

### 6.1 Magnetic nanoparticle-assisted optical aptasensors

Optical biosensors consist of a transducer that converts the interaction between a bioreceptor and target into a measurable optical signal and have found widespread application due to their sensitivity, simplicity, speed, and stability. Optical biosensors are classified based on the mechanisms of optical signal transduction into colorimetry, fluorescence, surface plasmon resonance, and surface-enhanced Raman scattering (SERS) biosensors (Shen et al., 2021).

#### 6.1.1 Magnetic nanoparticle-assisted colorimetric aptasensors

**Fig. 4:**
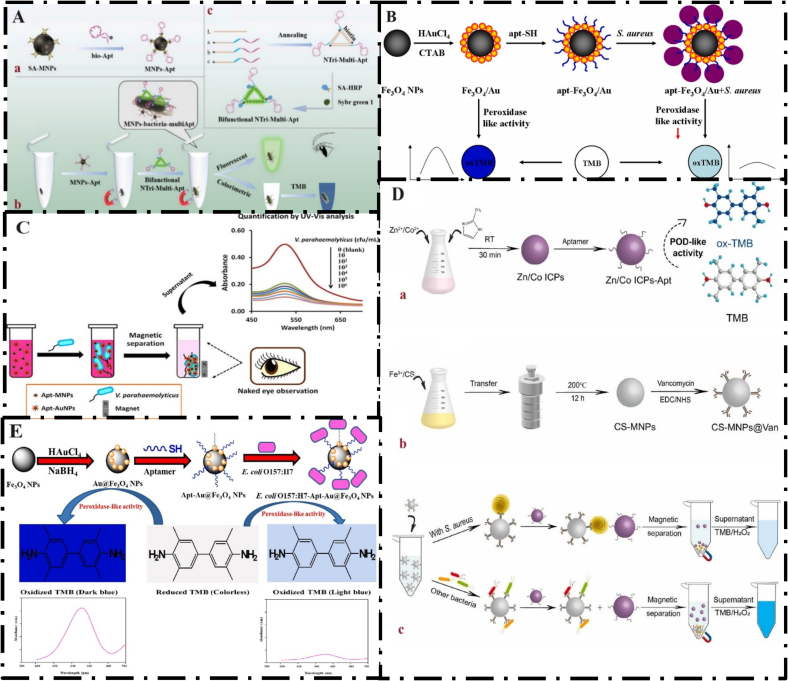
**(A)** An illustration of the preparation process for (a) MNP-Apts, (b) bifunctional NTri-Multi-Apts, and (c) the dual-mode detection of Salmonella utilizing colorimetric and fluorescence methods, which involves the magnetic separation of aptamers and NTri; From Ref. (4) with permission. (B) Schematic illustration of the synthesis process of Fe₃O₄/Au nanocomposites and colorimetric detection of *S. aureus*; From Ref. (7) with permission. (C) Schematic illustration of a colorimetric aptasensor for *V. parahaemolyticus* detection; From Ref. (11) with permission. (D) The preparation of (a) Zn/Co ICPs-Apt and (b) CS-MNPs@Van, (c) the detection of *S. aureus* based on Zn/Co ICPs-Apt and CS-MNPs@Van; From Ref. (15) with permission. (E) Steps for synthesis of Apt-Au@Fe_3_O_4_ nanoparticles and their utility for colorimetric detection of *E. coli* O157:H7; From Ref. (18) with permission.

The colorimetric method is a quantitative technique based on the relationship between the color change of a solution and the amount of a target substance. Colorimetric sensors have several advantages over other optical sensors, such as the ability to be integrated with portable platforms, visibility, low cost, and simple operation. These features have led to the widespread application of these platforms in various fields (Yang et al., 2024). The use of MNPs in colorimetric techniques has attracted much attention due to their unique properties. The target bacteria were effectively separated from the sample matrix via a magnet, which is a key advantage of the applied magnetic nanoparticles (see Section 5), prior to colorimetric measurement. MNPs exhibit higher catalytic stability than peroxidases when exposed to reaction conditions. MNPs possess intrinsic magnetic properties enabling enrichment and separation that make them suitable for biological applications. The synthesis and preparation of MNPs are simple and economical. However, the use of natural enzymes is costly and time-consuming (Zhang et al., 2010). Ma et al. developed a sensitive aptasensor for *Salmonella* detection. First, a nanotriangle multivalent aptamer was created, and a *Salmonella*-specific aptamer was immobilized on the surface of MNPs through biotin, forming the MNP-Apts complex (Fig. 4A). The interaction between biotin and streptavidin facilitated this process. Additionally, three DNA strands (modified with biotin a, b, and c) were used to create NTri-Multi-Apt nanotriangles, allowing for the simultaneous stabilization of the horseradish peroxidase molecule through biotin-streptavidin interactions. After the addition of SYBR Green I (an indicator of signal generation), a strong signal was produced due to the insertion of the indicator into the groove of the triangular dsDNA scaffold. In the presence of *Salmonella* (the target bacteria), separation occurred through the MNP-Apt complex, followed by NTri-Multi-Apt labeling, resulting in the formation of the MNPs-bacteria-multi-Apt complex, which was strongly fluorescent due to SYBR Green I embedded in the metal scaffold. On the other hand, in the presence of a chromogenic substrate and HRP catalysis, a color change (from colorless to dark blue) was observed, which can also be seen with the naked eye. The limits of detection (LOD) of this study were reported to be 316 CFU/mL and 60 CFU/mL for colorimetric and fluorescent methods, respectively (Fig. 4A) (Ma et al., 2023). Since even amounts as low as 10 CFU/g of *Salmonella* can cause pathogenicity, the use of sensitive and accurate diagnostic methods is of great importance. Although the method based on the synthesis of DNA nanotriangles has several advantages, this method has a high detection limit. Also, DNA nanotriangle synthesis requires thermal annealing and precise stoichiometry, which makes it somewhat more complicated than other colorimetric methods. Therefore, it requires optimization of reaction conditions and improvement of the nanotriangle design. Although Fe₃O₄ nanoparticles are most commonly used for separation, they have few active sites for binding to bioreceptors and may aggregate, reducing their magnetic properties. To overcome this problem and increase their efficiency and stability, these nanoparticles are coated with an Au shell (Kim & Yoo, 2022). Moreover, Zhang et al. developed a rapid colorimetric method for the detection of *S. aureus*. In this study, Fe₃O₄ nanoparticles were coated with gold nanoparticles, and then a specific thiol-modified aptamer was attached to the Fe₃O₄ /Au nanocomposites. In the presence of *S. aureus*, the formed apt-Fe₃O₄/Au nanocomposites bind to the bacterial surface. This process results in the preservation of their catalytic sites. The addition of H_2_O_2_ leads to the inhibition of the catalytic oxidation of TMB by the nanocomposites, and the color changes from dark blue to light blue in proportion to the bacterial concentration and appropriate catalytic activity (Fig. 4B). The advantages of this method include the absence of the need for washing and separation. The LOD, linear range, and detection time of this study were reported to be 10 CFU/mL, 10 to 10^6^ CFU/mL, and 12 min, respectively. This method was successfully tested on real samples such as milk and water, and its efficiency was confirmed (Zhang et al., 2021). Although specificity testing was performed in this study, it was not performed in the presence of other *Staphylococcus* species, such as *Staphylococcus epidermidis*, which could lead to interference in complex food samples. The performance of the proposed platform is highly dependent on pH conditions, which may not be possible in real samples. In another study, Sadsri et al. developed a sensitive and visual colorimetric method for the detection of *V. parahaemolyticus*. In this method, aptamer-functionalized MNPs (Apt-MNPs) and aptamer-functionalized gold nanoparticles were used as the specific separator and marker, respectively. In the presence of bacteria, Specific binding of the aptamer to bacteria (see Section 4) enabled the formation of the bacteria@Apt-MBs complex. Taking advantage of the optical properties of AuNPs allows for the observation of a visual signal and colorimetric detection without the need for an instrument. This simple and sensitive method was successfully applied to the detection of *V. parahaemolyticus* in contaminated raw shrimp samples. Also, the LOD and linear range of this study were reported to be 2.4 CFU/mL and 10–10^6^ CFU/mL, respectively (Fig. 4C) (Sadsri et al., 2020). However, AuNPs are prone to aggregation under unfavorable conditions (e.g., changes in salt, temperature, and pH) and can reduce the sensitivity of the sensor. Another essential issue in colorimetric assays is the complexity of food matrices. Food compositions contain polysaccharides, lipids, proteins, and other compounds, which leads to limited in situ detection of target analytes and interference in the analysis (Liu & Dong, 2023).

Zhou et al. developed a magnetic field-assisted colorimetric aptasensor for sensitive and selective detection of *S. aureus*. In this experiment, they used infinite coordination polymers (ICPs). ICPs are novel nanomaterials that are made by organic bridging ligands and metal ions. They have morphology-dependent properties, favorable compatibility, and flexible structure. Magnetic chitosan nanoparticles functionalized with vancomycin (CS-MNPs-Van) were used as the capture probe, and aptamer-labeled Zn/Co ICPs nanoenzyme (Zn/Co ICPs-Apt) were used as the sensing probe. The capture and separation of target bacteria from the complex food matrix were performed using a capture probe. To investigate the practical application of the designed colorimetric aptasensor, the amounts of *S. aureus* in real contaminated samples, such as seafood and bottled mineral water, were measured. In this study, magnetic enrichment of target bacteria from the food sample matrix was performed before the colorimetric signal output. Thus, it improves sensitivity and reduces interference. For this method, a linear range of 1.8 × 10^1^ - 1.8 × 10^5^ CFU/mL and LOD of 3 CFU/mL were reported (Fig. 4D) (Zhou et al., 2025). The authors state that the cost of the designed aptasensor is low and disposable, and that reuse of the proposed platform is not recommended due to cost, time, and human resources, which could pose problems in commercialization. In another study by Ali et al., a colorimetric sensor was designed for the detection of *E. coli* O157:H7. In this method, Au@Fe_3_O_4_ nanoparticles were first synthesized and then modified with a specific aptamer. In the presence of H_2_O_2_, the nanozymes used their peroxidase-like activity to convert reduced TMB to TMB oxide, which was accompanied by a color change from dark blue to light blue. The detection limit of this colorimetric aptasensor was reported to be 3 CFU/mL. It showed good performance for the detection of *E. coli* O157:H7 in milk, tap water, and orange juice samples (Fig. 4E) (Ali et al., 2024). In this study, the long-term stability of nanoparticles and aptamers was not investigated, and the sensor's performance in more complex food matrices, such as meat, eggs, and other foods, was not examined, which may limit the applicability of this platform.

#### 6.1.2 Magnetic nanoparticle-based fluorescence aptasensors

Fluorescence is a phenomenon in which an excited nanomaterial, molecule, or dye emits light after returning to its ground state (Majdinasab et al., 2018). Fluorescent sensors and assays have attracted much attention due to their speed, simplicity, high sensitivity, and practicality (Song et al., 2018). Metal-organic frameworks (MOFs) are a class of porous materials with numerous networks composed of metal nodes and organic ligands (as linkers) (Khezerlou et al., 2023). The combination of metal nanoparticles and metal-organic frameworks (MOF_S_) leads to the production of magnetic MOF_S_ that can be used as suitable adsorbents for the separation process. As a result, these adsorbents have some of the properties of magnetic materials and MOFs, such as rapid separation of the adsorbent from the solution, surface modification with functional groups, and high adsorption capacity at the same time (Ghiasi et al., 2022). For the development of sensors, MOFs can be designed to contain functional groups that bind to specific biological targets, thereby enhancing the selectivity of the sensor. Accordingly, Kumar et al. (2025) developed a fluorescent aptasensor using carboxyl-modified MNPs and a boric acid-functionalized terbium metal-organic framework (BA-Tb-MOF) for the detection of *Salmonella Typhimurium* (*S. typhimurium*) bacteria. To construct the capture probe, the MNPs were coated with a specific aptamer (Apt-MNPs), and the capture probe was added to the bacterial suspension to provide targeted binding with the bacteria. Subsequently, the fluorescent probe (BA-Tb-MOF) was added to the solution. Finally, the *S. typhimurium*-Apt-MNPs@BA-Tb MOF complex was formed. When the concentration of *S. typhimurium* in the medium increased. Due to the removal of *S. typhimurium*-Apt-MNPs@BA-Tb MOF complexes by magnetic separation, the fluorescence intensity gradually decreased. The LOD and linear range of this platform were reported to be 4 CFU/mL and 10–10^9^ CFU/mL, respectively. The designed platform successfully detected *S. typhimurium* in egg and drinking water samples. In this study, the unique properties of Fe_3_O_4_ MNPs, aptamers, and Tb-MOF were combined to develop a specific and sensitive platform. The integration of magnetic separation and fluorescence approaches led to the design of a rapid, sensitive, and selective method for the detection of *S. typhimurium* (Kumar et al., 2025). The specificity of this study was tested for a limited number of bacteria. Du et al. developed a specific and sensitive fluorescence-enhanced method for the detection of *Listeria monocytogenes* (*L. monocytogenes*). In this method, a combination of MOFs and MNPs was used. MOFs are suitable candidates for the immobilization of biomolecules due to their large surface area, diverse functional sites, and tunable pores. Magnetic Fe_3_O_4_ nanoparticles were used for magnetic separation and recovery.

**Fig. 5:**
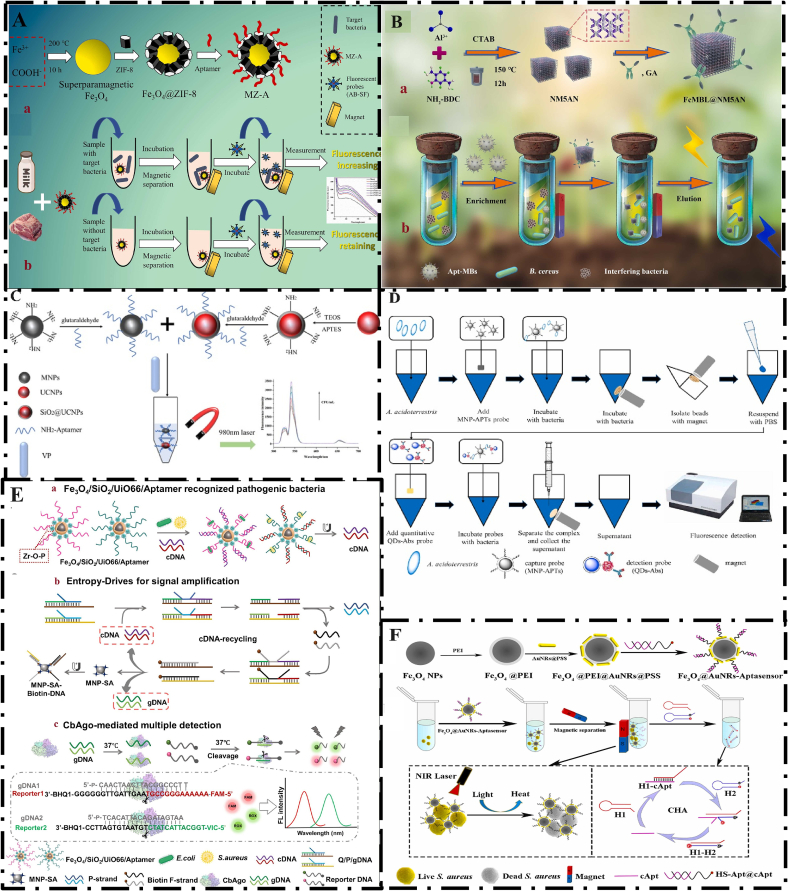
(A) Schematic illustrations of the preparation of Fe_3_O_4_@ZIF-8-aptamer and the detection system of L. *monocytogenes*; From Ref (66) with permission. (B) Synthesis of FcMBL@NM5AN and schematic description of the Detection of *B. cereus* using post-modified nano-MOF and aptamer; From Ref (67), with permission. (C) Schematic description of Detection of *V. parahaemolyticus* Based on Magnetic and Upconversion Nanoparticles Combined with Aptamers; From Ref. (70) with permission. (D) Schematic of the separation and detection of *A. acidoterrestris* by fluorescent QDs and aptamer-modified MNPs; From Ref. (72) with permission. (E) Magnetic MOF@aptamer-based Entropy-driven fluorescence biosensor combined with the CbAgo, the simultaneous detection of *S. aureus* and *E. coli*; From Ref (73) with permission.

The Fe_3_O_4_@ZIF-8 composite material used in this study exhibits both the advantages of Fe_3_O_4_ and ZIF-8. The aptamer was immobilized on Fe_3_O_4_@ZIF-8 to trap bacteria and then collected via magnetic confinement specifically. The linear range and LOD of this study were reported to be 1.4 × 10^1^ to 1.4 × 10^7^ CFU/mL and 0.88 CFU/mL, respectively. This system was successfully applied to detect L. *monocytogenes* in pork and milk samples and has high potential for application in diagnostic systems (Fig. 5A) (Du et al., 2022). To evaluate the specificity of the platform designed for the detection of L. *monocytogenes*, common foodborne pathogens, including *S. aureus*, *S. typhimurium*, *E. coli* O157:H7*, Pseudomonas aeruginosa*, and *V. parahaemolyticus*, were used. However, specificity was not evaluated for bacteria related to L. *monocytogenes*, which may reduce accuracy and generate false positives in real samples.

In another study, Yan et al. developed a dual strategy for the detection of *Bacillus cereus* (*B. cereus*). In this study, nano-MIL-53(Al)-NH2 modified with fragmented crystalline mannose-linked lectin was prepared as a fluorescent probe (FcMBL@NM5AN). Also, aptamer-coupled magnetic nanoparticles (Apt-MBs) were used as capture probes. In the presence of *B. cereus* bacteria in the medium, the specific aptamer bound to the target bacteria due to the high affinity of the aptamer, and the bactria@Apt-MBs complex was formed. Next, FcMBL@NM5AN was added to the reaction solution after magnetic separation by the magnet, and the fluorescence signal was detected. The LOD and linear range of this study were reported as 4 CFU/mL and 20 to 2 × 10^8^ CFU/mL, respectively. The developed aptasensor was satisfactorily used in the detection of *B. cereus* in food samples such as rice, eggs, poultry meat, distilled yeast, and milk (Fig. 5B) (Yan et al., 2023). Upconversion nanoparticles (UCNPs) can emit visible fluorescence under near-infrared light irradiation (NIR). As fluorescent labels, UCNPs have properties such as significant Stokes shift, low toxicity, blinking, photochemical degradation, and resistance to photobleaching, which lead to their superiority over traditional organic fluorophores (Fang et al., 2015). Using a magnetic field-assisted separation and concentration process, novel composite materials of aptamer-functionalized UCNPs and MNPs have been employed for the detection of foodborne pathogens (Jiang et al., 2017). Song et al. designed a fluorescence platform to detect *V. parahaemolyticus*. In this study, nanoparticles modified with aptamers were used to absorb *V. parahaemolyticus*. Also, aptamer-Fe_3_O_4_ and UCNP-aptamer were used as a separation medium and fluorescent signal, respectively. The basis of detection was based on aptamer target recognition (see Section 4), and separation was performed using magnetic nanoparticles. UCNP-aptamer and Bacteria-aptamer-Fe_3_O_4_ were combined to create sandwich seeds. The fluorescence intensity based on the presence of UCNPs was measured by spectrophotometry. The LOD and linear range of this study were reported as 4.4 CFU/mL and 3.2 × 10^2^ to 3.2 × 10^5^ CFU/mL, respectively. The results of this study showed that the proposed platform allows the detection of *V. parahaemolyticus* in different matrices (Fig. 5C) (Song et al., 2023). The limited linear range of the proposed platform can make its application in real examples challenging. Also, the stability of the nanoparticles used in this method may be affected by environmental factors such as temperature and pH, which could negatively affect the accuracy and reproducibility of the results. Fe_3_O_4_ nanoparticles are easily oxidized due to their small size. For this reason, Fe_3_O_4_ nanoparticles are coated with other materials. Silica (SiO_2_) is a material that is commonly used to coat nanoparticles. It is chemically inert, biocompatible, non-toxic, and has excellent thermal stability. The resulting Fe_3_O_4_@SiO_2_ core-shell MNPs have chemical stability, sensitive magnetic properties, and high potential for suitable surface modification. In addition, the silica coating on the surface of the nanoparticles prevents the aggregation of Fe_3_O_4_ nanoparticles in aqueous solution and increases their chemical stability (Bilgic & Cimen, 2020). Tian et al. (2021) used a combination of fluorescent quantum dots (QDs) and aptamer-modified MNPs for the isolation and detection of *Alicyclobacillus acidoterrestris* in fruit juices. In this study, a novel method was developed for the isolation and detection of *A. acidoterrestris* in apple juice samples. The core of this method was based on three key factors, including the high separation property of MNPs, the high sensitivity of fluorescent QDs, and the detection specificity of aptamers. Aptamer-functionalized MNPs were used for the rapid and specific detection of aptamers. Also, the specificity detection was performed using the fluorescent signal of antibody-based QDs, and finally, the complex (MNP-APTs- *A. acidoterrestris* -QDs-Abs) was formed. One potential challenge of this method is the possibility of interference of fluorescent signals between QDs and MNPs, which can reduce LOD. To overcome this limitation, a reverse strategy was used in this method, in which fluorescence detection was performed on unbound QDs present in the supernatant. The LOD and detection time of this study were reported to be 10^3^ and 90 min, respectively (Fig. 5D) (Tian et al., 2021). Among the limitations of this study are the high cost of the diagnostic platform due to the simultaneous use of aptamer and antibody. Additionally, there are complex steps for preparing and functionalizing nanoparticles. Lu et al. designed a Fe₃O₄@MOF@aptamer-based biosensor for non-proliferation detection of bacteria. In this study, entropy-mediated multiplex detection was performed without the need for DNA extraction and bacterial amplification. Argonautes (Agos) are abundant in prokaryotes and eukaryotes. Agos are a type of nucleic acid-directed endonuclease. Some Agos can cleave target nucleic acids by base pairing with nucleic acids. Magnetic Zr MOF nanoparticles (Fe₃O₄/SiO₂/UiO-66) were combined with phosphorylated aptamer and used as signal transducers. These nanoparticles recognize bacteria and release complementary DNA to activate an entropy-driven circuit reaction. Cb Ago (Argonaute derived from *Clostridium butyricum*) exerts precise and efficient endonuclease activity under the guidance of guide DNA. This process provides a biological platform for detection without DNA extraction and bacterial amplification. The detection limits of this system were reported to be 68 CFU/mL and 79 CFU/mL for *S. aureus* and *E. coli*, respectively. The biosensor was successfully tested on 20 chicken and 20 pork samples. The problems of this study include low sensitivity and poor stability in diagnosis. The authors suggest that future research should focus on modifying the structure of CbAgo using genetic engineering and protein modification technology to overcome these problems (Fig. 5E) (Lu et al., 2024). Extensive studies have been conducted in the field of pathogenic bacteria detection. However, the number of techniques that can simultaneously perform the process of detection and immediate control of pathogens is minimal. Accordingly, Kang et al. used a fluorescent platform for direct detection of *S. aureus* and its elimination. In this study, the elimination of bacteria was carried out by the photothermal properties of Fe_3_O_4_@AuNR under the influence of NIR excitation. This aptasensor was able to detect *S. aureus* in milk samples. The extensive advantages of this study include performing the enrichment, detection, and inactivation processes of bacteria simultaneously. The detection limit of this study was reported to be 10^2^ CFU/mL. The inactivation of pathogens is of great importance in the food industry. By improving this process, the developed platform could have widespread application in food safety control (Fig. 5F) (Kang et al., 2023).

#### 6.1.3 Magnetic nanoparticle-based surface-enhanced Raman scattering aptasensors

**Fig. 6:**
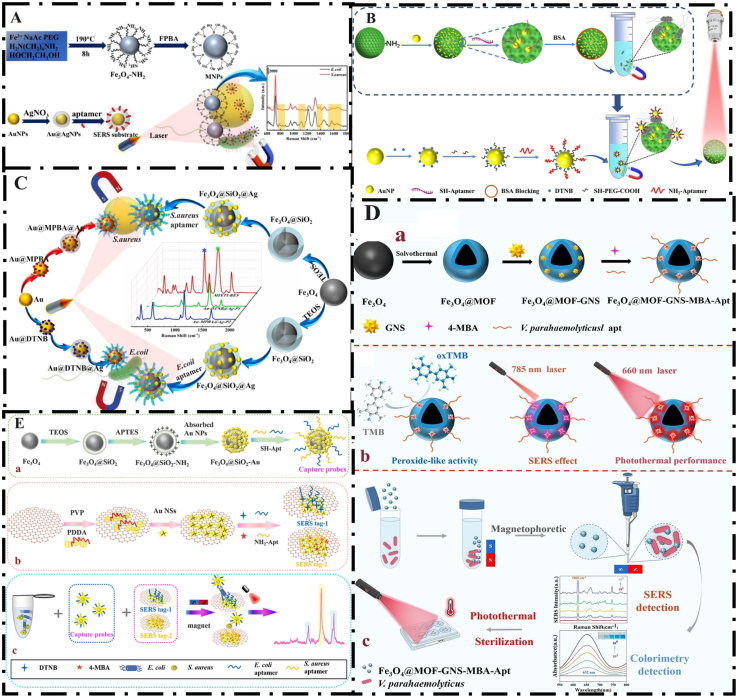
(A) Schematic of a label-free SERS biosensor for detection of pathogenic bacteria *S. aureus* and *E. coli*; From Ref (2) with permission. (B) Principle of the SERS-based detection platform for *E. coli* O157:H7; Reprinted from Ref. (8) with permission. (C) Schematic diagram of the dual-aptamer SERS biosensor for simultaneous detection of *S. aureus* and *E. coli*; From Ref. (12) with permission. (D) Schematic view of detection of *V. parahemolyticus* by colorimetric and SERS dual mode and photothermal sterilization; From Ref. (16) with permission. (E) Schematic of preparation of (a) Apt-Fe3O4@SiO2-Au magnetic capture probes, (b) aptamers conjugated SERS tags, and (c) dual strategy for simultaneously detecting *S. aureus* and *E. coli*; From Ref. (21) with permission.

Among the various methods introduced to detect pathogenic bacteria, the SERS method is significant due to its ease of accessibility, low background signal, non-destructive characteristics, and high sensitivity. Extensive studies have been conducted on the detection of pathogenic bacteria using the SERS method (Xiu et al., 2023). SERS biosensors can detect pathogenic bacteria in two ways: label detection and label-free detection. In the label detection method, Raman reporter molecules are usually used as SERS probes to label pathogenic bacteria and also increase the sensitivity of the detection method. While the label-free method allows for the direct recording of sample structural information, it typically requires a nearly pure background environment and statistical analysis in most cases. The label-free method is affected by the overlap of Raman signals of different reporters and the characteristics of the detection elements. Therefore, it is not suitable for the simultaneous detection of multiple pathogens. One of the main disadvantages of label-free SERS detection is that its sensitivity and reliability heavily rely on the SERS substrate, and complex samples can easily influence the method. To reduce the effect of interfering factors and quickly enrich and separate the target analytes, the use of magnetic SERS substrates as capture probes has been explored. Mi et al. developed a SERS biosensor for the simultaneous detection of *S. aureus* and *E. coli*. In this study, MNPs were functionalized with 4-formylphenylboric acid (FPBA). The boric acid group of FPBA can bind to the peptidoglycan layer on the surface of the bacterial cell wall and form stable cyclic esters in alkaline medium. Also, the reactions are reversed in acidic conditions. Therefore, by adjusting the pH, the magnetic materials can be recycled and regenerated. The SERS detection process was performed through the recognition of the aptamer target (see Section 4) by the modified Au@AgNPs. Aptamer-modified Au@AgNPs were used to enhance the Raman signal of bacteria. The advantages of this study include the excellent adsorption by FPBA, the recyclability of MNPs, and the formation of a SERS substrate with excellent performance due to the combination of two metals, Au and Ag. The detection limits of this study for *S. aureus* and *E. coli* were reported to be 18 CFU/mL and 34 CFU/mL, respectively. This diagnostic process was performed by a portable Raman spectrometer, which allows its use for point-of-care testing. The proposed platform was successfully tested on fresh shrimp samples (Fig. 6A) (Mi et al., 2023). However, designing a SERS-based diagnostic platform that performs optimally in terms of sensitivity, reproducibility, accuracy, and speed has always been a major challenge. The enhancement of SERS sensitivity is mostly dependent on the amplification of the electromagnetic hot spots (caused by the excitation of the plasmonic gold or silver surface by the incident light). The use of three-dimensional substrates leads to the creation of effective hot spots and results in a highly sensitive signal response. Three-dimensional substrates allow the uniform distribution of nanoparticles. Xiu et al. used the SERS detection method using a silica magnetic photonic microsphere array to detect *E. coli* O157:H7 in milk samples. It was based on combining aptamer-modified gold nanoparticles and macroporous silica magnetic photonic microspheres (Au@MMSPM). This system not only leads to rapid sample separation but also increases the sensitivity of the SERS method. The detection limit and linear range in this study were determined to be 2.20 CFU/mL and 10–10^6^ CFU/mL, respectively. The proposed platform is a rapid, sensitive, and accurate method for detecting *E. coli* O157:H7 and can be used in various fields of medicine and the food industry (Fig. 6B) (Xiu et al., 2023). Mi et al. developed a SERS biosensor combined with magnetic separation for the simultaneous detection of *E. coli* and *S. aureus*. Aptamer-modified Fe_3_O_4_@SiO_2_@AgNPs nanoparticles have strong magnetic properties and enable rapid enrichment and separation. This dual biosensor achieved a detection limit of 1 CFU/mL in milk samples within 20 min, demonstrating high sensitivity and versatility for the detection of various bacteria (Fig. 6C) (Mi et al., 2023). Li et al. used nanocomposites for photothermal sterilization and two-state colorimetric-SERS detection of *V. parahaemolyticus*. In this study, to synthesize the composite nanomaterials, firstly, the bimetallic node MOF-919 (Fe—Cu) (with peroxidase-like activity) was grown on the Fe_3_O_4_ surface, and Fe_3_O_4_@MOF was formed. Surface modification with thiol groups was performed to load gold sheets (GNS), and Fe₃O₄@MOF-GNS was prepared. Finally, 4-Mercaptophenylboronic acid (4-MBA) and a *V. parahaemolyticus*-specific aptamer were immobilized on the surface. As a result, multifunctional magnetic composite nanomaterials (Fe_3_O_4_@MOF(Fe—Cu)-GNS-MBA-Apt) were developed. When the bimetallic MOF was loaded, the composite material catalyzed TMB to oxTMB, and the color of the solution changed from colorless to blue. Colorimetric measurements were performed. Using GNS as a substrate and loading the 4-MBA molecule as a SERS signal, the composite material emitted SERS signals at a wavelength of 785 nm, enabling SERS detection. GNS has inherent photothermal conversion capability and generates heat under near-infrared radiation (660 nm), which enables rapid sterilization of the sample after detection. The dual-mode sensor showed a detection limit of 9 CFU/mL in the colorimetric method and 7 CFU/mL in the SERS method. The developed method showed high sensitivity, speed, and efficiency, and was successfully tested on fresh shrimp. The outstanding advantage of this study over other studies is that, in addition to pathogen detection, it also helps to improve the safety of the detection process and reduce the risk of secondary contamination in the detection environment (Fig. 6D) (Li et al., 2023). However, this method requires expensive equipment, and the process of making nanoparticles is complex. Plasmonic gold nanostars with sharp branches and favorable biocompatibility enable the generation of a large number of hot spots at their tips. They can exhibit stronger SERS enhancement than gold nanospheres or gold nanorods. The easy aggregation and poor stability of noble metal nanoparticles pose challenges in SERS detection. Graphene oxide (GO) has extensive advantages such as excellent dispersion stability, high biocompatibility, and a large specific surface area, and is an ideal support material for noble metal nanoparticles. The application of graphene oxide in SERS biosensors has extensive advantages, including inhibition of noble metal nanoparticle aggregation, quenching of fluorescent background signals, and improvement of detection sensitivity. Zhao et al. developed a dual SERS sandwich strategy for the simultaneous detection of *S. aureus* and *E. coli.* This strategy was based on aptamer-target binding (see Section 4) coupled with magnetic capture probes and SERS labels. The LOD of this study was reported to be 10 CFU/mL. This platform showed a lower LOD compared to other SERS biosensors and was used to detect *E. coli* and *S. aureus* in milk samples (Fig. 6E) (Zhao et al., 2023).

### 6.2 Magnetic nanoparticles-based electrochemical aptasensors

**Fig. 7:**
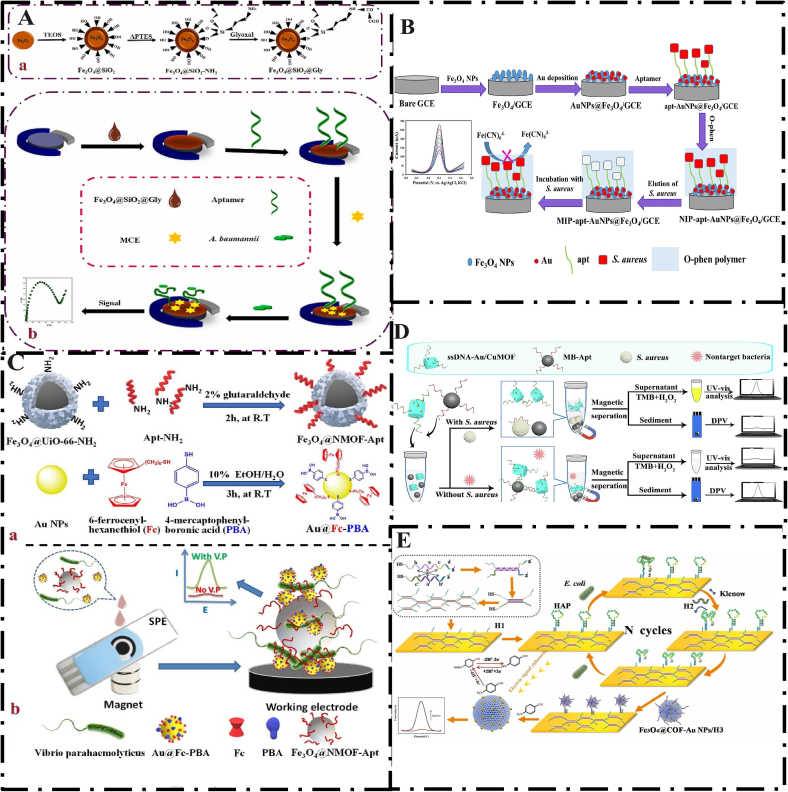
(A) Schematic illusion of (a) Synthesis steps of Fe_3_O_4_@SiO_2_@Glyoxal, (b) schematic illustration of the aptasensor fabrication and detection of *Acinetobacter baumannii* bacteria; From Ref.(1) with permission. (B) Schematic representation of a dual recognition aptasensor for specific detection of *S. aureus* based on Au/Fe_3_O_4_ binary hybrid; From Ref. (6) with permission. (C) Schematic illusion of (a) the preparation of nanolabel and capture probes, and (b) the signal-on type assay for *V. parahaemolyticus* on the SPE platform with magnet; From Ref.(10) with permission. (D) Illustration of electrochemical and colorimetric dual-mode detection of *S. aureus* based on a magnetic separation technique and multifunctional MOF; From Ref.(14) with permission. (E) Illustration of the construction of an electrochemical sensor by TICA and functionalized Fe_3_O_4_@COF-AuNPs; From Ref. (17) with permission.

Electrochemical biosensors are highly sensitive diagnostic platforms that convert biochemical signals into measurable electrical signals with the help of bioreceptors immobilized on the electrode surface. Thus, they enable quantitative and qualitative analyses of the target analyte. Electrochemical sensors have attracted much attention in recent years due to their extensive advantages, such as high efficiency, simplicity, and high sensitivity and specificity (Chang et al., 2022; Fatemi et al., 2024). The use of traditional solid electrodes to immobilize biomolecules, such as aptamers, enzymes, etc., presents many problems, for example, (a) the binding of biomacromolecules to the electrode surface can prevent the biomolecules from transferring. This process disrupts electron transport and reduces electrochemical signals. (b) The method of immobilizing biomolecules on the electrode surface is time-consuming. (c) Permanent immobilization of biological macromolecules on the electrode surface reduces the reproducibility of biosensors. To overcome these problems, MNPs and magnetic beads (MBs) were used as the solid phase in the design of electrochemical biosensors (Chang et al., 2022). The Fe₃O₄@COF-AuNPs nanocomposite was used to enhance the signal enhancement capability of magnetic nanoparticles (Section 5), which resulted in a significant enhancement of the electrochemical response (Wang et al., 2024). For example, Abedi et al. developed a sensitive aptasensor for the detection of *Acinetobacter baumannii* (*A*. b*aumannii*) using electrochemical impedance spectroscopy. To enhance the signal, a combination of SiO_2_, Fe_3_O_4,_ and glyoxal (Fe_3_O_4_@SiO_2_@Gly) was used. Functionalization of the surface of the nanocomposite (Fe_3_O_4_@SiO_2_) with Gly served two purposes. a) Formation of functional groups on the surface of the nanocomposite for binding to the NH_2_-aptamer, which led to increased specificity and sensitivity. b) to improve the colloidal stability of the nanocomposite in solution as a stabilizing agent. This simple platform has high selectivity, a simple operation process, and is suitable for in situ detection. The LOD and linear range of this study were reported to be 150 CFU/mL and 1 × 10^3^–1 × 10^6^ CFU/mL, respectively (Fig. 7A) (Abedi et al., 2023b). MNPs are used to separate target analytes in complex matrices using external magnetic fields. However, the poor electrical conductivity of MNPs limits their performance in electrochemical methods. Combining MNPs with gold nanoparticles facilitates rapid preconcentration and significantly enhances their conductivity (Zhao et al., 2020). El-Wekil et al. developed an electrochemical aptasensor for the detection of *S. aureus* based on Au/Fe₃O₄ binary nanohybrid. The use of Au/Fe₃O₄ binary nanohybrid resulted in superparamagnetic properties, low toxicity, excellent electrocatalytic properties, and improved electron transport and modification of the electrodes to increase conductivity. This platform was developed by immobilizing the aptamer on the surface of MNPs modified with gold nanoparticles (apt-AuNPs@Fe_3_O_4_). This designed aptasensor was made highly accurate, selective, and sensitive, and showed favorable results in the detection of *S. aureus* in real samples of apple juice, milk, and tap water. Other advantages of this aptasensor include low cost, high sensitivity, simplicity of operation and fabrication, very low detection limit (1 CFU/mL), and excellent selectivity (Fig. 7B) (El-Wekil et al., 2022). However, the process of constructing the proposed platform has multiple steps, which can make its practical use and commercialization difficult. The selectivity of the designed aptasensor against various pathogens has been investigated, but not against species closely related to *S. aureus*, such as *Staphylococcus epidermidis*, which can lead to false positive results. Also, to verify the validity of the proposed platform, this method should be compared with standard methods. UiO-66 is a nanoscale metal-organic framework (NMOF). NMOFs are an attractive group of crystalline materials that have great potential for use in biosensors, chemical separation, and drug delivery. Wang et al. developed an electrochemical aptasensor for the rapid detection of *V. parahaemolyticus* in seafood. In this study, aptamer-functionalized magnetic nanoscale metal-organic frameworks (Fe₃O₄@NMOF) were used as the capture probe, and gold nanoparticles with ferrocene and phenylboronic acid were used as the nanolabel (Au@Fc-PBA). After the probe/nanolabel was bound to the target, electrical signals were generated on the printed electrode surface. The magnetic nanolabels enable phase separation after enrichment and are also utilized in signal amplification. The developed aptasensor showed a low signal background without the need for purification or enrichment steps. The essential advantages of this platform include its reusability, portability, and on-site detection, making it an efficient method for bacterial screening and monitoring (Fig. 7C) (Wang et al., 2020). The designed aptasensor has a very high sensitivity (LOD 3 CFU/mL), a wide linear range (10–10^9^ CFU/mL), and a very short detection time (20 min), which enables rapid detection and in-situ usability for the designed platform. The disadvantages of the present study are the lack of comparison of the proposed aptasensor with standard methods and the lack of investigation of the selectivity of the developed platform against species such as *V. vulnificus* or *V. cholerae*. Chen et al. used a colorimetric and electrochemical dual-mode aptasensor for the detection of *S. aureus*. In this study, they used an ssDNA-Au/CuMOF as the source and signal amplifier. Due to the accumulation of high amounts of chelated copper and the remarkable catalytic performance of Au/CuMOF compared to the 3,3′,5,5′-Tetramethylbenzidine substrate, strong electrochemical and color signals were observed. The use of a dual output method enhances detection accuracy, resistance to interferences in complex food samples, and good selectivity. Other advantages of this method include its efficient and convenient application owing to the use of magnetic beads. The feasibility of the developed aptasensor was confirmed in complex food materials such as orange juice, milk, and tap water. The detection limit of the designed aptasensor was 5 CFU/mL, and the linear range was 10 to 10^8^ CFU/mL (Fig. 7D) (Chen et al., 2023). The developed platform has a very low detection limit and a wide linear range, which is competitive or even superior to other electrochemical aptasensors. One of the most important strengths of this study is the dual-mode detection (electrochemical and colorimetric), which increases the reliability of the experiment. However, the preparation and execution of the experiment are time-consuming, which can pose challenges for the commercialization of the proposed aptasensor. To enhance the saturation magnetism of MNPs, pure metal particles such as cobalt, iron particles, or other types of MNPs, such as CuFe₂O₄, CoFe₂O₄, and NiFe₂O₄, which contain smaller amounts of copper, cobalt, and nickel, are used. However, MNPs doped with heteroatoms have limitations. For example, nickel and cobalt are prone to oxidation and are highly toxic. As a result, limited research has been conducted on them. Covalent organic frameworks (COFs) are a group of emerging porous materials that have been widely used in sensor development due to their regular pore structure, large surface area, low density, and tunable performance. On the other hand, Fe_3_O_4_ nanoparticles have attracted much attention due to their redox properties, high electrochemical response, and low toxicity. Combining COFs with MNPs leads to the integration of the advantages of both and increases the sensitivity of the sensor. In addition, COFs prevent the aggregation of AuNPs due to their porous structure, and their catalytic performance is significantly enhanced when COFs are combined with Fe_3_O_4_ nanoparticles (Yang et al., 2023a). Accordingly, Zhang et al. developed a highly sensitive electrochemical biosensor based on trigging isothermal circular amplification (TICA) and magnetic Fe_3_O_4_@COF-AuNPs nanocomposite. The Fe_3_O_4_@COF-AuNPs nanocomposite, due to its large surface channels, allowed for the regular arrangement of AuNPs, which led to increased contact with the substrate (4-nitrophenol) and increased redox signal. This platform provided a novel approach for the early detection of *E. coli* bacteria. The detection limit of this biosensor was reported to be 10 CFU/mL, and the detection time was 1 h. The recovery rate was 92.0 % to 109.0 % in food samples such as milk, orange juice, and human serum, which demonstrates the reliability of this developed platform for the detection of *E. coli* in real samples (Fig. 7E) (Zhang et al., 2025). The low detection limit, narrow linear range, and short detection time make this electrochemical sensor very suitable for diagnostic processes and clinical applications. Commercialization of this platform requires simplification of design and synthesis. The designed aptasensor was successfully tested on food samples such as milk. However, its performance in complex samples such as meat has not been investigated. The complex matrix of this food can interfere with the performance of the biosensor. Electrochemical aptasensors have presented promising approaches for the detection of pathogens. Magnetofluidic systems are a promising platform for simplifying complex diagnostic platforms. The use of magnetic bead nanoparticles in the structure of biosensors, due to quantum effects, high surface area, and superparamagnetic properties, allows for the appropriate preconcentration of target biomolecules. In recent years, magnetic-microfluidic devices have been developed for the electrochemical detection of pathogens in food samples. Although these platforms have yielded significant results, performing the measurement process in a closed system, due to the presence of a mixture of solutions, allows for the possibility of system contamination. Panphut et al. developed sensitive and accurate electrochemical aptasensing for the detection of *Salmonella* in food samples. The designed aptasensing operates via a microfluidic system and consists of a superhydrophobic surface made of polydimethylsiloxane (through a gold chemical reduction process). This system allows for control of fluid orientation and distinct magnetic flux and prevents sample mixing. Thus, sample contamination is minimized. The detection limit of this study was reported to be 10 CFU/mL, and the detection time was 30 min. The designed platform was successfully tested on food samples such as chicken, pork, high-fat meat, shrimp, and pork (Panphut et al., 2025). In addition to the aforementioned advantages, the aptasensor designed for commercialization needs to improve the long-term stability of Apt-MB. Although the proposed platform has been investigated in various food products, the authors point out that for high-fat samples, the pre-processing process may require adjustment and optimization to eliminate interfering compounds. Machine learning is an emerging technology that has the potential to extract information from large and complex data. Integrating machine learning into electrochemical sensors can bring many benefits. Electrode fouling, variable operating conditions, and degradation of biological components can lead to the formation of nonlinearities in the aptasensor signal. Machine learning can overcome these limitations. Machine learning can also combine electrochemical data with other data methods, ultimately improving the prediction performance (Bocan et al., 2025). Dong et al. designed a diagnostic aptasensor based on the combination of Au@Apt-functionalized MNPs and the application of a machine learning model. This accurate and sensitive aptasensor was used to detect *E. coli* in Yakult and tap water samples. The Fe_3_O_4_@Au@Apt complex was formed through the Au—S bond between Fe_3_O_4_@Au nanoparticles and the aptamer, and the efficient capture and separation of the target bacteria were achieved. To accurately predict the bacterial concentration, an XGBoost model was built using time-frequency features for the aptasensor. The results of this study reported a linear range of 10–10^7^ CFU/mL and the LOD of 1 CFU/mL. Also, the intelligent diagnostic framework showed excellent prediction performance with a mean absolute error of 0.087 CFU/mL and an R2 of 0.990 (Dong et al., 2025). The key advantages of this study include the combination of nanomaterials and machine learning. The very low detection limit and wide detection range highlight the applicability of this developed aptasensor for diagnostic purposes. However, model optimization requires expertise, specialized devices, and software, limiting its application in the field. The developed aptasensor was successfully tested for food materials such as Yakult and drinking water, but it was not investigated for food materials with complex matrices.

### 6.3 Aptamer-based other assays

**Fig. 8:**
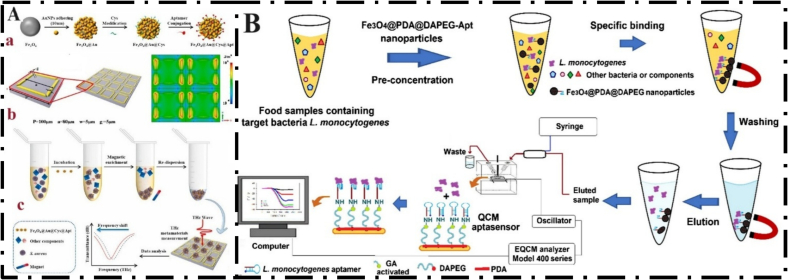
(A) Detection of *S. aureus* using a THz metamaterial biosensor: (a) Synthesis of Fe_3_O_4_@Au@Cys@Apt, (b) Schematic of a structure unit of the THz metamaterials and electric field distribution at the resonant frequency, (c) THz metamaterial biosensor for *S. aureus* detection; From Ref. (3) with permission. (B) Synthesis of the Fe_3_O_4_@PDA@DA-PEG particles; From Ref. (5) with permission**.**

Terahertz (THz) spectroscopy, as an emerging biosensing technology, reveals structural information of biomolecules based on long-range and intermolecular vibrational modes. This technique has been applied in various bacterial studies due to its ability to provide label-free and rapid detection of bacteria based on their distinct molecular components (Yu et al., 2024, Zhou et al., 2021a). THz metamaterials are composed of subwavelength metallic resonators. These synthetic electromagnetic materials are susceptible to compounds deposited on their surfaces and exhibit changes in refractive index or permittivity, ultimately leading to changes in resonant frequency. As mentioned, multifunctional MNPs have demonstrated broad utility in the field of biosensors. These nanoparticles enable the separation of the target analyte from complex environmental solutions, reduce the detection time, and eliminate the need for sample pretreatment. However, MNPs have a high refractive index. To overcome this problem, MNPs were combined with THz metamaterials to improve detection sensitivity. Yu et al. designed a THz metamaterial biosensor that utilizes functionalized nanomaterials for the rapid detection of *S. aureus*. In this study, an *S. aureus-*specific aptamer was attached to the Fe₃O₄@Au nanocomposite using L-cysteine to form the final structure Fe₃O₄@Au@Cys@Apt. In the presence of the target bacteria, this nanocomposite specifically binds to *S. aureus* cells. This process leads to its selective enrichment in complex environmental samples and enhances the detection sensitivity through THz signal amplification. The LOD of this biosensor is reported to be 3.0 × 10^2^ CFU/mL, and its linear range of operation is up to 5.0 × 10^7^ CFU/mL. The main advantages of this method are high speed, simple design, and label-free detection (Fig. 8A) (Yu et al., 2024). Bayramoglu et al. developed a novel diagnostic platform for L. *monocytogenes* detection in chicken meat and milk samples. In this study, a bacterial-specific aptamer was selected by the SELEX method, and by combining the aptamer and magnetic separation system, an innovative method was designed for sensitive and rapid separation of L. *monocytogenes* from liquid and solid food samples. The magnetic system in the proposed platform enables fast and efficient separation of L. *monocytogenes* from the medium with the help of a small adsorbent. For this purpose, Fe_3_O_4_ nanoparticles were first coated with polydopamine (PDA) and then grafted with diamino-polyethylene glycol (DA-PEG), resulting in the formation of two biocompatible and hydrophilic layers on the surface of the particles. The aptamer was immobilized on the MNPs with separation achieved through aptamer-target recognition (see Section 4). Therefore, Fe_3_O_4_@PDA@DAPEG-Apt particles were used for the separation of L. *monocytogenes*. The trapped target bacteria were identified by plate counting. The traditional culture method requires 2 to 6 days. The time needed for this study without the use of expensive devices has been reported to be 18 h, while the time required for electrochemical methods varies between 1 and 4 h. However, the proposed advantages of this system include the lack of expensive equipment, simplicity, easy regeneration, low cost, repeated use, and rapid isolation of target bacteria. The detection limit of this study was reported to be 1.4 × 10^2^ CFU/mL and was suitably used for food isolation (Fig. 8B) (Bayramoglu et al., 2024).

## 7 Challenges and perspectives of magnetic nanoparticles-assisted aptasensors for foodborne pathogen monitoring

Food safety is a critical aspect of public health. Foodborne microorganisms threaten the health of a large number of people worldwide every year. Therefore, the development of a rapid and reliable diagnostic method for the detection of these bacterial pathogens is essential. Aptamer-based sensors need extensive improvements to become a standard diagnostic tool in the food industry. Aptamers are oligonucleotide sequences that are extracted from the SELEX library in vitro. These oligonucleotide sequences are superior to antibodies due to their high affinity for the target molecule, stability under various temperature conditions, and ability to bind to small molecules. However, they are vulnerable to harsh environmental conditions and enzymatic degradation. The affinity of aptamers for their targets can be improved through structural modifications. One of these approaches is the addition of nucleobases such as 7-(2-thienyl) imidazo[4,5-b] pyridine, which can increase the specific interaction between the aptamer and the target molecule. Also, the addition of aliphatic or aromatic functional groups at the 5-position of the deoxyuridine triphosphate (dUTP) nucleotide can help increase the affinity of aptamers (Abedi et al., 2025). Another effective method is the use of modified versions of the SELEX process, such as Cell-SELEX and in vivo-SELEX, which enable the selection of aptamers under real or physiological conditions. Therefore, future studies should focus more on the design of aptamers. In this context, the use of artificial intelligence to predict the interactions between aptamers and target bacteria can greatly contribute to increasing their affinity, and further research in this area is necessary (Wong et al., 2025). In recent years, extensive progress has been made in the field of electrochemical aptasensors based on MNPs for the detection of pathogenic bacteria (Table 2). However, the number of these studies is less compared to colorimetric, fluorescent, and SERS methods. Given the broad advantages of these methods, they could be a good field for future research. A number of foodborne microorganisms, such as *Shigella*, can be pathogenic even at very low levels, which highlights the high sensitivity of the diagnostic methods. These problems can be caused by several challenges, such as the difficulty of designing a specific aptamer with high sensitivity and a very low detection limit for some of these bacteria. Future research should focus on further studies on optimizing the use of MNPs in the structure of biosensors. The combination of molecular methods and aptasensors, given the high potential of both methods, can lead to the improvement and increase of the sensitivity of diagnostic platforms. The application of magnetic nanoparticle-based aptasensors for detection has been largely limited to the laboratory setting. These aptasensors have a long way to go before they can be commercialized. Among the challenges to commercializing these methods is the difficulty of preparing magnetic nanoparticles with uniform size and shape on a large scale. Also, signal readout usually requires bulky and expensive devices, which makes it difficult to analyze bacteria in situ. To overcome these problems, future studies should focus on integrating these platforms with commercially available aptasensors to develop their scalable synthesis. Multiplex detection of bacteria in food using magnetic aptasensors is recognized as an innovative and accurate approach in the field of food safety. However, existing studies on the application of this technology are still limited, and its further development requires more comprehensive research. On the other hand, these methods are time-consuming, their design is complex, they may negatively affect the specificity of aptamers, and they also require special analytical equipment for sample analysis. To overcome these limitations, aptamers with specific labels can be used and combined with magnetic nanoparticles, especially with other nanoparticles that have unique optical and electrochemical properties. This combination can significantly improve the diagnostic performance. The application of such methods, especially in the food industry, is of great importance to prevent microbial contamination and ensure consumer health. Another challenge for aptasensors is the development of portable diagnostic equipment for the rapid detection of pathogenic bacteria, especially in resource-limited environments. These devices play an effective role in improving food safety by reducing the cost and time of detection. However, their entry into commercial markets requires overcoming challenges such as achieving high sensitivity, reducing LOD, ensuring good reproducibility, long-term stability, and efficiency in sample preparation in complex food matrices. Although biosensors have extensive advantages, improving their reproducibility and stability has always been a challenge. The application of machine learning in future studies can improve the overall performance and reliability of the biosensor system. The use of nanomaterials in a biosensor can reduce the signal-to-noise ratio and help a suitable learning algorithm. In addition, the possibility of developing a multidisciplinary biosensor platform with high efficiency and sensitivity, portability and miniaturization for the detection of foodborne pathogens and further monitoring of food safety has been investigated. In the future, the development of aptasensors attached to magnetic nanoparticles is expected to take very important steps towards addressing key challenges in the field of pathogen detection. In this regard, future research should work more on increasing the sensitivity and selectivity of these aptasensors. Advances in the synthesis, design and engineering of magnetic nanoparticles play an important role in this field. The integration of aptasensors with artificial intelligence, machine learning and bioinformatics is one of the future directions in the development of magnetic nanoparticle-based aptasensors. To increase the sensitivity of these sensors, optimizing the sensor design and operating conditions, including the use of artificial intelligence algorithms to interpret the data, can improve the overall sensor performance (Hassan et al., 2025). Among the reviewed studies, electrochemical and SERS methods showed the highest sensitivity. A number of studies did not report the detection time. In this comparison, colorimetric methods had the shortest detection time. Although methods such as SERS also showed short detection times, sample preparation in these methods may be time-consuming. In terms of cost, colorimetric methods are less expensive compared to other methods, while electrochemical systems require electrical signal measurement equipment. Also, all the methods used were successfully tested on food samples. Despite the extensive progress in the development of aptasensors for the detection of foodborne pathogens, a review of recent studies shows that these investigations have mostly focused on a few specific bacteria such as *Salmonella*, *E. coli*, *L. monocytogenes* and *S. aureus*, highlighting the urgent need for comprehensive investigations in future studies. This limitation could be due to several challenges, such as designing specific aptamers with high sensitivity and achieving very low LOD for some of these bacteria. To overcome this problem, the use of MNPs in the structure of biosensors and the combination of molecular methods and aptasensors have been proposed to improve and increase the sensitivity of diagnostic platforms. Electrochemical aptasensors provide fast response times and are cost-effective compared to other sensing technologies. In recent years, studies have been conducted on magnetic nanoparticle-based electrochemical aptasensors for the detection of pathogenic bacteria; however, these studies are far fewer than those of colorimetric, fluorescent, and SERS methods. Given the extensive advantages of these methods, they can be a field for future research. One of the methods for synthesizing MNPs is green synthesis. This method is of great importance because it does not use hazardous reagents and toxic solvents. Green synthesis is environmentally friendly. It reduces the cost of producing nanoparticles and is a suitable alternative to traditional synthesis methods. However, this method is associated with limitations such as the need to optimize various reaction parameters (temperature, pH, and reaction time). The exact mechanism of green synthesis of nanoparticles for use in biosensors remained unknown. Future research should focus more on improving synthesis methods, leading to the production of stable, environmentally friendly magnetic nanoparticles and nanoparticles with more precise surface areas (Carinelli et al., 2023; Samuel et al., 2022). The application of green-synthesized MNPs in the structure of biosensors for detecting environmental pollutants has been very limited. This provides a good opportunity for future research in this area. Future research could focus more on the development of magnetic nanoparticle-assisted aptasensors integrated with smartphones. These smartphone-based biosensors enable on-site monitoring, rapid testing, powerful data analysis, and easy operation. Given the large population of mobile phone users, the development of these biosensors could revolutionize the detection of foodborne bacteria (Abdelbasset et al., 2023). However, several challenges need to be addressed, such as the high cost of aptamer reagents, interference from complex food matrices, and aptamer instability under different environmental conditions. Another challenge for the application of MNPs in biosensor structures is their environmental issues. The release and accumulation of nanoparticles in the ecosystem is a threat to aquatic and terrestrial life. Currently, a regulatory framework for the accurate assessment of the environmental impact of these nanoparticles has not been reported. To overcome these challenges, several solutions have been proposed, including the following: a) Establishing joint working groups between the WHO–FAO Codex committees to harmonize international guidelines. b) Establishing innovation centers that allow academia, regulatory agencies, and industry to develop standardized protocols for the characterization of nanoparticles. c) Conducting a life-cycle risk assessment of nanoparticles (from production to disposal) allows monitoring of environmental safety and sustainability (Jangid et al., 2025).

**Table 2:** Overview of magnetic nanoparticle-assisted aptasensors for foodborne bacteria monitoring.

**Methods**	**Target bacteria**	**Detection limit** **(CFU/mL)**	**Detection time** **(min)**	**Real Sample**	**Ref.**
**Optical**					
Color	*L. monocytogenes*	14	80	Milk and chicken breast	(Du et al., 2024b)
Color	*L. monocytogenes*	10	50	Pork	(Wang et al., 2022)
Color	*E. coli O157:H7*	0.2549	120	Milk	(Zhang et al., 2022)
Color	*L. monocytogenes*	12	90	Chicken breast and milk	(Du et al., 2024a)
Color	*E. coli*	0.987	–	Milk, grape juice, and orange juice	(Li et al., 2024)
FL	*Campylobacter and Aliarcobacter*	1	65	Chicken meat	(Liu et al., 2024)
FL	*Salmonella*	138	–	Lettuce	(Guo et al., 2020b)
FL	*E. coli*	10	–	Pork	(Li et al., 2020)
FL	*L. monocytogenes*	10	–	Fresh water and pork meat	(Guo et al., 2020a)
FL	*B. cereus*	22	–	Milk	(Zheng et al., 2022)
SERS	*S.aureus* *L. monocytogenes*	3.5	90	Orange juice and extracts of lettuce	(Cheng et al., 2021)

**Methods**	**Target bacteria**	**Detection limit** **CFU/mL**	**Detection time** **(min)**	**Real Sample**	**Ref.**
SERS	*S. typhimurium*	5	–	Pork	(Ma et al., 2016)
SERS	*S. typhimurium*	4	–	Chicken and milk	(Yang et al., 2021)
SERS	*E. coli* *L. monocytogenes* *S. typhimurium*	10 10 25	–	Milk and extracts of lettuce	(Zhou et al., 2021b)
SERS	*S. aureus*	96	–	Milk	(Ma et al., 2021)
SERS	*S. aureus*	25	30	Milk	(Zhao et al., 2022)
SERS	*S. aureus*	1.09	50	Milk, tap water, and orange juice	(Qi et al., 2022)

Electrochemical					
Electrochemical	*A. baumannii*	1	–	Skim milk powder	(Abedi et al., 2023a)
Electrochemical	*E. coli*	6.88	–	Milk and water	(Lin et al., 2021)
Dual-mode aptasensor					
Color/FL	*S. aureus*	Color (20) FL (22)	>40	Meat	(Ouyang et al., 2021)
Color/SERS	*Campylobacter jejuni*	6	15	Water	(Zhao et al., 2024)
Color/FL	*E. coli*	Color (10) FL (6)	–	Milk	(Huang et al., 2024)

Abbreviations: Color, Colorimetric; Fl, fluorescence; SERS, surface-enhanced Raman scattering.

## 8 Conclusion

In this study, recent advances in magnetic nanoparticle-assisted aptasensors for foodborne bacteria monitoring using optical, electrochemical, and other methods were reviewed. Foodborne pathogens are one of the major causes of illness and death worldwide. Given the widespread concerns regarding food safety, the rapid, sensitive, accurate, and in situ detection of foodborne bacteria is of great importance for ensuring public health and hygiene. The application of nanomaterials in the structure of biosensors is carried out for various purposes, such as the generation, conversion, and amplification of signals, purification, immobilization of target biomarkers, and separation. This leads to reduced response time, improved performance, the detection of analytes at lower concentrations, and high reproducibility and stability of nanosensors. However, many of the developed methods require method improvement to achieve lower detection limits. These goals can be achieved by increasing the affinity of aptamers through modifying their chemical structure, using modified versions of SELEX, using artificial intelligence, and combining diagnostic methods such as molecular and aptasensory methods; therefore, they can be topics for future research. Also, the complex food matrix can lead to interference in the diagnostic field. Although the use of MNPs for magnetic enrichment of target bacteria from the food sample matrix has been widely used, there are many challenges in this regard. However, the high cost of the materials and equipment used, the high detection limit of some of them, reproducibility, and improving their stability are still raised as challenges. Another challenge in the use of MNPs is the issue of their accumulation and disposal in the environment, which can pose risks to the ecosystems and requires the coordination of international regulations and the conduct of a life cycle risk assessment of nanoparticles. Despite extensive studies in the field of MNP-based biosensors for detecting foodborne bacteria, the application of MNPs in aptasensor structures is limited to the laboratory environment, and commercialization of these methods requires optimization of MNP synthesis methods, integration of diagnostic methods, and a focus on intelligent platform design to overcome these challenges, which can be attractive topics for future studies.

## CRediT authorship contribution statement

**Narges Kiani-Salmi:** Writing – original draft. **Behnam Bahramian:** Writing – original draft, Supervision. **Reza Abedi-Firoozjah:** Software, Investigation. **Alireza Ebrahimi:** Validation, Project administration. **Arezou Khezerlou:** Formal analysis. **Milad Tavassoli:** Supervision. **Ali Ehsani:** Supervision.

## Declaration of competing interest

The authors declare that they have no known competing financial interests or personal relationships that could have appeared to influence the work reported in this paper.

## Data Availability

The data that has been used is confidential.

## Appendix A Supplementary data

Supplementary material. Image 1
