# Supplementary material for: Recent advances and trends in magnetic nanoparticle-assisted aptasensors for foodborne bacteria monitoring: applications, challenges, and updates
Source: Food Chem X. 2025 Oct 16;31:103156. doi: 10.1016/j.fochx.2025.103156 (PMC12593589; doi:10.1016/j.fochx.2025.103156)
Supplement: Supplementary file 1 — Supplementary material. [file mmc1.docx]

**Supplementary Material**

**Recent advances and trends in** **magnetic nanoparticle-assisted aptasensors for Foodborne Bacteria Monitoring: Applications, challenges, and updates**

Narges Kiani-Salmi^1,2†^, Behnam Bahramian^1,2†^, Reza Abedi-Firoozjah^3†^, Alireza Ebrahimi^1,2^, Milad Tavassoli^4^, Arezou Khezerlou^1^, Ali Ehsani^1, 5^**^*^**

^1^ Department of Food Science and Technology, Faculty of Nutrition and Food Sciences, Tabriz University of Medical Sciences, Tabriz, Iran

^2^ Student Research Committee, Tabriz University of Medical Sciences, Tabriz, Iran

^3^ Student Research Committee, Kermanshah University of Medical Sciences, Kermanshah, Iran

^4^ Department of Nutrition, Faculty of Health and Nutrition Sciences, Yasuj University of Medical Science, Yasuj, Iran

^5^ Nutrition Research Center, Tabriz University of Medical Sciences, Tabriz, Iran

*Corresponding author

Email: [ehsani@tbzmed.ac.ir](mailto:ehsani@tbzmed.ac.ir)(Ali Ehsani)

**^†^**Equal first author.

S.1 Fabrication and Properties of Magnetic Nanoparticles

## **1. Chemical Methods**

Some of these methods, direct application in sensors, have not been reported to date, and they could have potential for future studies in sensor development. This may be the case.

### **1.1. Chemical Co-precipitation (CCP)**

Co-precipitation is a simple and common method that is widely used to synthesize various types of nanoparticles. This method requires an aqueous medium for precipitation. This method was used to fabricate Fe_3_O_4_ nanoparticles in a non-enzymatic electrochemical glucose sensor structure. The results of this study reported a detection limit of 0.03 mM and a detection time of 3s (Carinelli et al., 2023). He et al. used a co-precipitation method to synthesize magnetic nanoparticles (MNPs). The synthesized MNPs were conjugated with a complementary DNA of T-2 aptamer (cDNA) and used as adsorption probes for the detection of T-2 toxin. The detection limit of this study was reported to be as low as 0.035 ng/mL (He et al., 2019).

**1.2 Hydrothermal method**

Another method for synthesizing MNPs is the hydrothermal technique. This technique uses a high-pressure and high-temperature reactor with an aqueous solution as the medium. In this context, Ozel and colleagues reported that by increasing the reaction time from 1 h to 120 h, the average particle size increased from 14 ± 4 nm to 74 ± 9 nm (Ozel and Kockar, 2015). In another study, Tadic et al. found that the saturation magnetization of MNPs also increased with increasing temperature and reaction time (Tadic et al., 2019). Researchers synthesized nanocrystalline nickel spinel ferrites (NiFe_2_O_4_) using a hydrothermal technique and glycerol (surfactant), which showed nanoparticles of uniform size without aggregation (Dinkar et al., 2018). Luo et al. used a hydrothermal method to synthesize magnetic core-shell hollow carbon spheres (Fe_3_O_4_@MHCS). The synthesized magnetic nanoparticles were utilized to fabricate an electrochemical sensor for detecting organophosphate pesticides. The detection limit of this study was reported to be 0.0182 ppb (Luo et al., 2018). MNPs are used for drug delivery and biomolecule delivery due to their high bioconjugation efficiency. Yadav et al. used a hydrothermal method to synthesize Fe_3_O_4_ nanoparticles. In this study, the interaction between Calf thymus (Ct) DNA and MNPs was investigated. All the experiments performed demonstrated a change in DNA conformation due to the interaction, which could facilitate numerous applications in the construction of magnetic nanoparticle-based biosensors and drug delivery systems (Yadav et al., 2020)

### **1.3 Thermal decomposition**

This technique produces high-purity metal elements or compounds by heating the metal oxide. For example, for synthesizing Fe_3_O_4_ MNPs, an organic compound containing iron is added to a hot solution with surfactants, which decomposes and oxidizes iron atoms at high temperatures, and Fe_3_O_4_ MNPs are produced (Patsula et al., 2016). Annealing temperature influences the magnetic properties and size of MNPs. For example, at an annealing temperature of 300°C, the mean particle size distribution of superparamagnetic MNPs was 7.38 ± 1.9 nm, while MNPs at annealing temperatures of 900 and 700°C showed ferromagnetism (Amara et al., 2009). Studies have shown that ligands, metal precursors, and innovative synthesis processes affect physical properties (Vuong et al., 2020, Ibrahim et al., 2018, Sartori et al., 2019). The researchers reported that they produced controllable monodisperse MNPs at 305°C with Fe(III) acetylacetonate as the starting precursors (Sun et al., 2004). Compared with the CCP technique, thermal decomposition has advantages in monodispersity, controllable size, and narrow size distribution. However, this method has disadvantages, such as surface spreaders and the high cost of metal-organic complexes, which limit its use on a large scale (Li et al., 2023). Cobalt ferrite nanoparticles exhibit properties such as a magnetic anisotropy constant and coercivity at room temperature, which have enhanced their application in biosensor structures. By using the thermal decomposition method of iron-cobalt oleate precursor in a high-boiling solvent, cobalt ferrite nanoparticles with a narrow size distribution can be obtained (Herrera et al., 2013).

### **1.4. Polyol Method**

This technique is the simplest method for the synthesis of MNPs. It facilitates the synthesis of MNPs from inorganic compounds, including sulfides, alloys, fluorides, and oxides (Ruz and Sudarsan, 2021). In this technique, polyols are a reducing agent, a polar organic solvent for metal precursors, and a complexing agent for metal cations (Roy, 2022). Khan et al. developed a novel voltammetric sensor for the determination of vitamin B_9_ in food. Magnetic molecular imprinted polymers (MMIPs) were synthesized using the polyol method. The developed biosensor was successfully developed for the detection of folate in food samples such as oranges and broccoli (Khan et al., 2022).

**1.5. Sol–Gel Method**

This wet chemistry technique forms gels at 25°C through the multiple condensation reactions of metal alkoxides and hydrolysis. Metal salts can dissolve in water or other solvents to form a colloidal solution or sol (Ansari et al., 2019). In this method, van der Waals forces are induced between the MNPs, and increasing the temperature and stirring increases the interaction between the nanoparticles (Stiufiuc and Stiufiuc, 2024). The MNPs synthesis mixture is heated until the solvent evaporates, and the solution eventually dries and forms a gel (Ali et al., 2021).

### **1.6. Microemulsion**

This technique uses a thermodynamic mixture containing oil, water, and surfactants to synthesize MNPs. After thermal treatment, the microbubbles nucleate and aggregate, and MNPs are produced in the emulsion system (Nguyen et al., 2020). This technique forms a protective layer during the synthesis of MNPs, which reduces aggregation and modification of MNPs. Different sizes of MNPs are obtained by controlling the total number of microemulsions. In addition, the aggregation, magnetic interaction, and stabilization of MNPs limit their application because they significantly reduce the reaction site and specific surface area. Microemulsion can solve the problems mentioned above by encapsulating MNPs in layers, such as a silica layer and liposomes (Salvador et al., 2021, Nitica et al., 2022).

### **1.7. Pyrolysis Methods**

This technique involves forming tiny droplets in precursor solutions during the synthesis process that are sprayed in a specific high-temperature environment. The solutes obtained after solvent evaporation are subjected to chemical reaction processes to form the desired material. The advantages of this technique include homogeneous coating, controllable particle size, scalability, tunable magnetic properties, and versatility of MNPs (Tahir et al., 2020). The laser pyrolysis technique utilizes CO_2_ laser radiation to induce chemical reactions in a precursor material, ultimately resulting in the production of nanoparticles. The principle is that a high-intensity laser beam is focused onto the precursor material in the aerosol or gas phase, and the laser beam's energy is absorbed by the precursor, causing it to heat up and decompose. The resulting chemical processes produce nanoparticles, which are then collected on the MNPs substrate (Wang and Gao, 2019).

## **2. Physical Methods**

### **2.1. Lithography**

This versatile method has been used to synthesize various types of nanoparticles, including MNPs. This technique is part of the micropatterning technique based on the controlled transfer of patterns from a template or mask to a substrate. It allows for the precise placement and arrangement of nanoscale features. This method has been optimized in recent years, and new techniques have been used, including scanning, electron beam, colloidal lithography, and nanosphere (Habibullah et al., 2021).

### **2.2. Mechanical Milling**

This technique converts coarse particles into nanoparticles by mechanical grinding using a top-down approach. A hollow-centered cylindrical glass mill converts the precursor material to micro/nano size. This method is limited by the production of particles of different sizes and the possibility of product contamination (Stiufiuc and Stiufiuc, 2024).

### **2.3. Laser Evaporation**

This technique, also known as vapor deposition or laser ablation, is a physical method classified under the bottom-up approach. This technique forms nanoparticles by a condensation process from the gas or liquid phase. High-energy laser beams are used to vaporize raw materials, which are then cooled to a gaseous state, causing rapid condensation and nucleation, ultimately leading to the formation of nanoparticles (Stiufiuc and Stiufiuc, 2024).

### **2.4. Wire Explosion**

This technique is a new method for the synthesis of MNPs that is clean and safe and does not require additional steps such as treating residues or separating nanoparticles from the solution. It has been studied for the production of magnetic iron oxide nanoparticles for use in the removal of arsenic from water (Kawamura et al., 2015).

### **2.5. Gas-Phase Synthesis**

This technique is classified as the bottom-up approach and has been used to synthesize multifunctional nanoparticles. These nanoparticles are produced using a gaseous precursor that provides control over size, crystallinity, and composition. The two techniques for gas phase synthesis include inert gas condensation and chemical vapor condensation. The chemical vapor condensation technique reduces metal precursors in the gas phase (Stiufiuc and Stiufiuc, 2024). Chemical precursors such as carbonyls and metal halides are used for this technique, which, at high temperatures, cause the reduction of these precursors, leading to the production of MNPs. In the inert gas condensation technique, MNPs are produced by condensation and subsequent cooling of an inert gas resulting from the vacuum evaporation of metal clusters or atoms (Hammad et al., 2020).

## **3. Biological Methods**

Plants and microorganisms are recognized as new sources with high potential for nanoparticle synthesis. Biological methods for nanoparticle synthesis offer several advantages, such as reproducibility in production, non-toxicity, well-defined morphology, and easy scalability. So far, plants and microorganisms (including fungi, bacteria, and yeasts) have been used for nanoparticle synthesis (Singh et al., 2016).

### **3.1. Synthesis by Microorganisms**

The synthesis of nanoparticles by microorganisms is a green technology. Various types of prokaryotic and eukaryotic microorganisms are used for the synthesis of metal nanoparticles such as iron, platinum, gold, zinc oxide, titanium oxide, and others. Magnetotactic bacteria can synthesize intracellular magnetic particles, including iron sulfides, iron oxides, or both. The proteins of bacterial magnetic particles, arranged in chains inside the bacteria, act as biological compass needles and help the bacteria navigate aquatic environments along oxygen gradients. Jou et al. synthesized mesoporous Fe_3_O_4_ magnetic materials from yeast cells using a co-precipitation method (Hasan, 2015, Li et al., 2011). Synthesis of nanorods with microorganisms increases the efficiency of metal ion reduction and provides conditions for the synthesis of magnetic nanorods. Magnetobacteria are famous for their ability to synthesize MNPs intracellularly. Fungi have been documented for synthesizing iron oxide nanoparticles, which hold bright prospects in remediation and biomedicine (Gul et al., 2019). Microbial methods provide a sustainable and environmentally friendly route for the synthesis of MNPs. These methods not only allow precise control of the morphology and size of nanoparticles but are also highly reproducible, cost-effective, and sustainable. The production of large quantities of nanoparticles using bacterial fermentation is very advantageous because it provides high production rates, easy recovery, and consistent particle sizes. Compared to chemical synthesis methods, microbial methods have lower energy consumption and operate at lower temperatures. Therefore, they are a promising approach for large-scale industrial applications. (Moon et al., 2010, Rudayni et al., 2025).

### **3.2. Synthesis with plant extracts**

Phytochemicals have been widely utilized in the synthesis of MNPs. Plant extracts are used as stabilizers and reducing agents. Different parts of plants, such as stems, leaves, and roots, have been used to extract bioactive compounds. For example, green tea and aloe vera extracts have been successfully used to synthesize iron oxide nanoparticles. Nanoparticles have shown promise in magnetic resonance imaging and drug delivery (Zhang et al., 2019, Stiufiuc and Stiufiuc, 2024). Kobylinska et al. employed an eco-friendly and efficient method to biosynthesize CoFe₂O₄ and Fe₃O₄ nanoparticles using root extracts from the hairy root culture of *Artemisia annua L*. These extracts, rich in phytochemicals, helped reduce Fe (II, III) and Co (II) ions into magnetic nanoparticles like magnetite and cobalt ferrite. The nanoparticles were then used to remove heavy metal ions from water (Kobylinska et al., 2021). Mabarroh et al. designed a biosensor using magnetic nanoparticles to label biomolecules. For this study, magnetic nanoparticles were synthesized by a green synthesis method. The results of this study showed that the green-synthesized Fe_3_O_4_ nanoparticles exhibited no significant differences in morphology, magnetic properties, and crystal structure compared to the original Fe_3_O_4_ nanoparticles. The results of this study showed that green-synthesized MNPs are a novel method for magnetic labeling in biosensors (Mabarroh et al., 2022).

**3.3. Synthesis with enzymes**

One of the emerging advances in nanotechnology is the synthesis of nanoparticles using enzymes. Many enzymes derived from microbes and plants have been utilized due to their ability to synthesize metallic nanoparticles. However, limited studies have reported the molecular and biochemical mechanisms of nanoparticle synthesis using enzymes. Enzymes can exhibit different roles during nanoparticle formation, such as acting as capping and reducing agents. They increase the reaction rate but do not participate directly in biochemical reactions (Sanket and Das, 2021).

For the first time, Kolhatkar and colleagues successfully synthesized magnetic nanoparticles enzymatically in the laboratory. This achievement introduced a novel approach for the development of magnetic sensors. In this process, magnetic nanoparticles were synthesized in situ using enzymes, which can overcome limitations related to mass transfer. The produced nanoparticles exhibited unique magnetic properties, including antiferromagnetism, paramagnetism, and a strong saturation magnetization. Applying this method in magnetic sensors resulted in a signal enhancement of up to 10,000 times compared to optical-based sensing techniques (Kolhatkar et al., 2015).

**Table 1.** Different fabrication methods for MNPs.

| **Type** | **Description** | **Advantages** | **Disadvantages** |
| --- | --- | --- | --- |
| **[A] Chemical Methods** | | | |
| Chemical Co-precipitation | Iron hydroxide is oxidized and precipitated in alkali solutions. | Quick synthesis, industrial scalability possible. | Poor control over particle properties and shape. After the reactants are mixed, MNPs form rapidly, leading to the disruption of process control |
| Hydrothermal Method | Wet chemical technique in a high-pressure, high-temperature environment. | Good uniformity and magnetic response; suitable for specific shapes. | Time-consuming and costly; requires high safety measures.  The magnetic and structural properties of MNPs depend on temperature, surfactant content. |
| Thermal Decomposition | Heating metal oxides to produce pure metallic nanoparticles. | High purity, monodispersity, and narrow size distribution. | High cost of materials, scalability issues. |
| Polyol Method | Involves polyols as solvents and reducing agents to synthesize various MNPs. | Simple, environmentally friendly, cost-effective. | Potentially limited to specific types of nanoparticles. |
| Sol–Gel Method | Formation of gels through the condensation of metal alkoxides. | Well-defined shapes and controllable sizes of nanoparticles. | Requires careful control of gelation and drying processes. |
| Microemulsion | Uses thermodynamic mixtures of oil, water, and surfactants to synthesize nanoparticles. | Reduces aggregation and provides control over particle size. | Complication in scale-up and potential for toxicity from surfactants. |
| Pyrolysis Methods | Synthesis through the thermal decomposition of precursors in high-temperature environments. | Homogeneous coating, tunable properties, and good scalability. | High cost and potential technical challenges in scalability. |
| **[B] Physical Methods** | | | |
| Lithography | Micropatterning technique that transfers patterns from a template to a substrate. | High resolution and precise spatial control of nanoparticles. | Complex setup and generally high costs. |
| Mechanical Milling | Top-down method for reducing particle size through mechanical grinding. | Simple equipment and straightforward process. | Contamination and size variability issues. |
| Laser Evaporation | Uses high-energy lasers to vaporize materials, resulting in nucleation and condensation of nanoparticles. | Cost-effective and minimal hazardous waste. | Requires advanced technology and precise conditions. |
| Wire Explosion | A safe, clean method for synthesizing nanoparticles without further processing steps. | Environmentally friendly; straightforward production process. | Non-uniform distribution of nanoparticles may limit applications. |
| Gas-Phase Synthesis | Techniques like inert gas condensation and chemical vapor condensation to synthesize nanoparticles. | Controlled particle size and composition. | High equipment costs and complexity in implementation. |
| **[C] Biological Methods** | | | |
| Synthesis by Microorganisms | Utilizes fungi, bacteria, and viruses to synthesize nanoparticles. | High efficiency and potential for complex structures. | Limited control over synthesis conditions and possible toxicity. |
| Synthesis with Plant Extracts | Employs phytochemicals from plants as stabilizers and reducing agents for nanoparticle synthesis. | Simple, natural, and cost-effective methodology. | Variability based on plant source composition. |
| Synthesis with Enzymes | Enzymatic catalysis to produce controlled, uniform nanoparticles. | High precision and biocompatibility for medical applications. | Limited to specific nanoparticles; may require complex setups. |

**References**

ALI, A., SHAH, T., ULLAH, R., ZHOU, P., GUO, M., OVAIS, M., TAN, Z. & RUI, Y. 2021. Review on recent progress in magnetic nanoparticles: Synthesis, characterization, and diverse applications. *Frontiers in chemistry,* 9**,** 629054.

AMARA, D., FELNER, I., NOWIK, I. & MARGEL, S. 2009. Synthesis and characterization of Fe and Fe3O4 nanoparticles by thermal decomposition of triiron dodecacarbonyl. *Colloids and Surfaces A: Physicochemical and Engineering Aspects,* 339**,** 106-110.

ANSARI, S. A. M. K., FICIARÀ, E., RUFFINATTI, F. A., STURA, I., ARGENZIANO, M., ABOLLINO, O., CAVALLI, R., GUIOT, C. & D’AGATA, F. 2019. Magnetic iron oxide nanoparticles: synthesis, characterization and functionalization for biomedical applications in the central nervous system. *Materials,* 12**,** 465.

CARINELLI, S., LUIS-SUNGA, M., GONZÁLEZ-MORA, J. L. & SALAZAR-CARBALLO, P. A. 2023. Synthesis and modification of magnetic nanoparticles for biosensing and bioassay applications: a review. *Chemosensors,* 11**,** 533.

DINKAR, D. K., DAS, B., GOPALAN, R. & DEHIYA, B. S. 2018. Effects of surfactant on the structural and magnetic properties of hydrothermally synthesized NiFe2O4 nanoparticles. *Materials Chemistry and Physics,* 218**,** 70-76.

GUL, S., KHAN, S. B., REHMAN, I. U., KHAN, M. A. & KHAN, M. 2019. A comprehensive review of magnetic nanomaterials modern day theranostics. *Frontiers in Materials,* 6**,** 179.

HABIBULLAH, G., VIKTOROVA, J. & RUML, T. 2021. C urrent strategies for noble metal nanoparticle synthesis. *Nanoscale Research Letters,* 16**,** 47.

HAMMAD, M., HARDT, S., MUES, B., SALAMON, S., LANDERS, J., SLABU, I., WENDE, H., SCHULZ, C. & WIGGERS, H. 2020. Gas-phase synthesis of iron oxide nanoparticles for improved magnetic hyperthermia performance. *Journal of Alloys and Compounds,* 824**,** 153814.

HASAN, S. 2015. A review on nanoparticles: their synthesis and types. *Res. J. Recent Sci,* 2277**,** 2502.

HE, D., WU, Z., CUI, B., XU, E. & JIN, Z. 2019. Building a fluorescent aptasensor based on exonuclease-assisted target recycling strategy for one-step detection of T-2 toxin. *Food Analytical Methods,* 12**,** 625-632.

HERRERA, A. P., POLO-CORRALES, L., CHAVEZ, E., CABARCAS-BOLIVAR, J., UWAKWEH, O. N. & RINALDI, C. 2013. Influence of aging time of oleate precursor on the magnetic relaxation of cobalt ferrite nanoparticles synthesized by the thermal decomposition method. *Journal of magnetism and magnetic materials,* 328**,** 41-52.

IBRAHIM, E., ABDEL-RAHMAN, L. H., ABU-DIEF, A. M., ELSHAFAIE, A., HAMDAN, S. K. & AHMED, A. 2018. Electric, thermoelectric and magnetic characterization of γ-Fe2O3 and Co3O4 nanoparticles synthesized by facile thermal decomposition of metal-Schiff base complexes. *Materials Research Bulletin,* 99**,** 103-108.

KAWAMURA, G., ALVAREZ, S., STEWART, I. E., CATENACCI, M., CHEN, Z. & HA, Y.-C. 2015. Production of oxidation-resistant Cu-based nanoparticles by wire explosion. *Scientific Reports,* 5**,** 18333.

KHAN, S., WONG, A., RYCHLIK, M. & SOTOMAYOR, M. D. P. T. 2022. A novel synthesis of a magnetic porous imprinted polymer by polyol method coupled with electrochemical biomimetic sensor for the detection of folate in food samples. *Chemosensors,* 10**,** 473.

KOBYLINSKA, N., KLYMCHUK, D., SHAKHOVSKY, A., KHAINAKOVA, O., RATUSHNYAK, Y., DUPLIJ, V. & MATVIEIEVA, N. 2021. Biosynthesis of magnetite and cobalt ferrite nanoparticles using extracts of “hairy” roots: preparation, characterization, estimation for environmental remediation and biological application. *RSC advances,* 11**,** 26974-26987.

KOLHATKAR, A. G., DANNONGODA, C., KOURENTZI, K., JAMISON, A. C., NEKRASHEVICH, I., KAR, A., CACAO, E., STRYCH, U., RUSAKOVA, I. & MARTIROSYAN, K. S. 2015. Enzymatic synthesis of magnetic nanoparticles. *International journal of molecular sciences,* 16**,** 7535-7550.

LI, W., XIAO, F., BAI, X. & XU, H. 2023. Magnetic nanoparticles for food hazard factors sensing: synthesis, modification and application. *Chemical Engineering Journal,* 465**,** 142816.

LI, X., XU, H., CHEN, Z.-S. & CHEN, G. 2011. Biosynthesis of nanoparticles by microorganisms and their applications. *Journal of nanomaterials,* 2011**,** 270974.

LUO, R., FENG, Z., SHEN, G., XIU, Y., ZHOU, Y., NIU, X. & WANG, H. 2018. Acetylcholinesterase biosensor based on mesoporous hollow carbon spheres/core-shell magnetic nanoparticles-modified electrode for the detection of organophosphorus pesticides. *Sensors,* 18**,** 4429.

MABARROH, N. M., ALFANSURI, T., WIBOWO, N. A., ISTIQOMAH, N. I., TUMBELAKA, R. M. & SUHARYADI, E. 2022. Detection of green-synthesized magnetite nanoparticles using spin-valve GMR-based sensor and their potential as magnetic labels. *Journal of Magnetism and Magnetic Materials,* 560**,** 169645.

MOON, J.-W., RAWN, C. J., RONDINONE, A. J., LOVE, L. J., ROH, Y., EVERETT, S. M., LAUF, R. J. & PHELPS, T. J. 2010. Large-scale production of magnetic nanoparticles using bacterial fermentation. *Journal of Industrial Microbiology and Biotechnology,* 37**,** 1023-1031.

NGUYEN, C.-C., GANDON, A. & DO, T.-O. 2020. Novel Route to Preparing Magnetic Fe3O4@ SiO2@ MoO3 Core–Dual Shell Nanoparticles via Solid-Phase Reverse Microemulsion for the Oxidative Cleavage of Fatty Acids. *ACS Applied Nano Materials,* 3**,** 10571-10577.

NITICA, S., FIZESAN, I., DUDRIC, R., BARBU-TUDORAN, L., POP, A., LOGHIN, F., VEDEANU, N., LUCACIU, C. M. & IACOVITA, C. 2022. A Fast, Reliable Oil-In-Water Microemulsion Procedure for Silica Coating of Ferromagnetic Zn Ferrite Nanoparticles Capable of Inducing Cancer Cell Death In Vitro. *Biomedicines,* 10**,** 1647.

OZEL, F. & KOCKAR, H. 2015. Growth and characterizations of magnetic nanoparticles under hydrothermal conditions: Reaction time and temperature. *Journal of Magnetism and Magnetic Materials,* 373**,** 213-216.

PATSULA, V., KOSINOVÁ, L., LOVRIĆ, M., FERHATOVIC HAMZIĆ, L., RABYK, M., KONEFAL, R., PARUZEL, A., ŠLOUF, M., HERYNEK, V. & GAJOVIĆ, S. K. 2016. Superparamagnetic Fe3O4 nanoparticles: synthesis by thermal decomposition of iron (III) glucuronate and application in magnetic resonance imaging. *ACS applied materials & interfaces,* 8**,** 7238-7247.

ROY, I. 2022. Therapeutic applications of magnetic nanoparticles: recent advances. *Materials Advances,* 3**,** 7425-7444.

RUDAYNI, H., ALLAM, A. A., SUR, D., BALLAL, S., SHUKRI, S. M., TANTAWI, D. A., RAY, S. & SHANKHYAN, A. 2025. Tailored Magnetic Nanoparticles for High-Performance Detection and Remediation of Heavy Metal Pollutants in Aquatic Environments. *Journal of Molecular Structure***,** 143805.

RUZ, P. & SUDARSAN, V. 2021. Polyol Method for synthesis of nanomaterials. *Handbook on Synthesis Strategies for Advanced Materials: Volume-I: Techniques and Fundamentals***,** 293-332.

SALVADOR, M., GUTIÉRREZ, G., NORIEGA, S., MOYANO, A., BLANCO-LÓPEZ, M. C. & MATOS, M. 2021. Microemulsion synthesis of superparamagnetic nanoparticles for bioapplications. *International journal of molecular sciences,* 22**,** 427.

SANKET, S. & DAS, S. K. 2021. Role of enzymes in synthesis of nanoparticles. *Bioprospecting of enzymes in industry, healthcare and sustainable environment***,** 139-153.

SARTORI, K., CHOUEIKANI, F., GLOTER, A., BEGIN-COLIN, S., TAVERNA, D. & PICHON, B. P. 2019. Room temperature blocked magnetic nanoparticles based on ferrite promoted by a three-step thermal decomposition process. *Journal of the American Chemical Society,* 141**,** 9783-9787.

SINGH, P., KIM, Y.-J., ZHANG, D. & YANG, D.-C. 2016. Biological synthesis of nanoparticles from plants and microorganisms. *Trends in biotechnology,* 34**,** 588-599.

STIUFIUC, G. F. & STIUFIUC, R. I. 2024. Magnetic Nanoparticles: Synthesis, Characterization, and Their Use in Biomedical Field. *Applied Sciences,* 14**,** 1623.

SUN, S., ZENG, H., ROBINSON, D. B., RAOUX, S., RICE, P. M., WANG, S. X. & LI, G. 2004. Monodisperse mfe2o4 (m= fe, co, mn) nanoparticles. *Journal of the American chemical society,* 126**,** 273-279.

TADIC, M., TRPKOV, D., KOPANJA, L., VOJNOVIC, S. & PANJAN, M. 2019. Hydrothermal synthesis of hematite (α-Fe2O3) nanoparticle forms: Synthesis conditions, structure, particle shape analysis, cytotoxicity and magnetic properties. *Journal of Alloys and Compounds,* 792**,** 599-609.

TAHIR, M. B., RAFIQUE, M., RAFIQUE, M. S., NAWAZ, T., RIZWAN, M. & TANVEER, M. 2020. Photocatalytic nanomaterials for degradation of organic pollutants and heavy metals. *Nanotechnology and Photocatalysis for Environmental Applications.* Elsevier.

VUONG, T. K. O., LE, T. T., DO, H. D., NGUYEN, X. T., NGUYEN, X. C., VU, T. T. & LE, T. L. 2020. PMAO-assisted thermal decomposition synthesis of high-stability ferrofluid based on magnetite nanoparticles for hyperthermia and MRI applications. *Materials Chemistry and Physics,* 245**,** 122762.

WANG, S. & GAO, L. 2019. Laser-driven nanomaterials and laser-enabled nanofabrication for industrial applications. *Industrial applications of nanomaterials.* Elsevier.

YADAV, N., SINGH, A. & KAUSHIK, M. 2020. Hydrothermal synthesis and characterization of magnetic Fe3O4 and APTS coated Fe3O4 nanoparticles: physicochemical investigations of interaction with DNA. *Journal of Materials Science: Materials in Medicine,* 31**,** 68.

ZHANG, Q., YANG, X. & GUAN, J. 2019. Applications of magnetic nanomaterials in heterogeneous catalysis. *ACS Applied Nano Materials,* 2**,** 4681-4697.
